# ﻿A revision of the endemic Brazilian *Solanumhexandrum* group (Leptostemonum, *Solanum*, Solanaceae)

**DOI:** 10.3897/phytokeys.253.138216

**Published:** 2025-03-12

**Authors:** Sandra Knapp, Yuri F. Gouvêa, Leandro L. Giacomin

**Affiliations:** 1 Natural History Museum, Cromwell Road, London SW7 5BD, UK Natural History Museum London United Kingdom; 2 Departamento de Botânica, Instituto de Ciências Biológicas, Universidade Federal de Minas Gerais, Av. Antônio Carlos 6627, Belo Horizonte 31270-901, MG, Brazil Universidade Federal de Minas Gerais Belo Horizonte Brazil; 3 Departamento de Sistemática & Ecologia, Centro de Ciências Exatas e da Natureza, Universidade Federal da Paraíba, Cidade Universitária, João Pessoa, PB, 58051-0900, Brazil Universidade Federal da Paraíba João Pessoa Brazil

**Keywords:** Atlantic Forest, Brazil, endemism, fruit morphology, prickles

## Abstract

The Leptostemonum Clade, or the ‘spiny solanums’, represents half of the species diversity of the large cosmopolitan genus *Solanum* (Solanaceae). Brazil is a centre of both species and lineage diversity in ‘spiny solanums’ with a number of lineages occurring mostly only there. Here, we treat the *Solanumhexandrum* group, a monophyletic species group that is part of the larger and unresolved Erythrotrichum clade *sensu lato.* The six species treated here are all robust very prickly shrubs with amongst the largest and showiest flowers in *Solanum* and accrescent calyces in fruit that often completely cover the mature berry. All six species are endemic to the coastal Atlantic forests of south-eastern and north-eastern Brazil. We describe one new species, *S.phrixothrix* Gouvêa & S.Knapp, **sp. nov.**, known only from two collections made 200 years apart. Many of the species in the group occur in very small populations around isolated gneissic/granitic inselbergs, a highly threatened habitat in the region. We provide complete nomenclatural details for all recognised species and their synonyms, complete descriptions, distributions including maps, illustrations, common names and uses and preliminary conservation assessments.

## Abstract (es)

O clado Leptostemonum, ou dos ‘solanums aculeados’, representa metade da diversidade de espécies do hiperdiverso e cosmopolita gênero *Solanum* (Solanaceae). O Brasil é um centro de diversidade de espécies e linhagens de *Solanum* aculeados, com uma série de linhagens ocorrendo principalmente no país ou restritas a ele. Aqui apresentamos o tratamento do grupo *Solanumhexandrum*, uma linhagem que faz parte do clado maior e ainda não resolvido, chamado de *Erythrotrichumsensu lato.* As seis espécies tratadas aqui são todas arbustos robustos e armados, algumas portando flores entre as maiores e mais vistosas de *Solanum*, com cálices tipicamente acrescentes que muitas vezes cobrem completamente os frutos maduros. Todas as seis espécies são endêmicas das Floresta Atlântica brasileira, ocorrendo nas regiões Sudeste e Nordeste do país. Descrevemos uma nova espécie, *S.phrixothrix* Gouvêa & S.Knapp, **sp. nov.**, conhecida apenas por duas coletas feitas com 200 anos de diferença. Muitas das espécies do grupo ocorrem em populações muito pequenas ao redor de inselbergs de graníticos e gnáissicos isolados, um habitat altamente ameaçado na região. Fornecemos detalhes nomenclaturais completos para todas as espécies reconhecidas e seus sinônimos, descrições, informação sobre sua distribuição, incluindo mapas, ilustrações, nomes comuns e usos, e avaliação preliminar de seu *status* de conservação.

## ﻿Introduction

*Solanum* L. is one of the ten most species-rich genera of flowering plants (Frodin 2004; Moonlight et al. 2024), with 1,245 currently accepted species occurring on all temperate and tropical continents (Hilgenhof et al. 2023). The highest diversity of both groups and species is in tropical South America, concentrated around the Amazon Basin (see Knapp (2002)), with hotspots of species diversity in the north-central Andes and south-eastern Brazil. Species of *Solanum* have flowers with fused sepals and petals that are usually 5-merous, stellate to pentagonal to rotate or somewhat campanulate corollas, stamens with short filaments and anthers opening by terminal pores. Despite this apparent uniformity, the genus as currently recognied has not always been treated as a monophyletic group (Spooner et al. 1993; Bohs 2005). Phylogenetic reconstruction using DNA sequence data has shown that previously segregated genera such as *Lycopersicon* Mill. and *Cyphomandra* Sendtn. (amongst others) are part of a larger monophyletic *Solanum* with strong support (Weese and Bohs 2007; Särkinen et al. 2013; Gagnon et al. 2022). Thirteen major clades are recognised in *Solanum* (see Gagnon et al. (2022); Hilgenhof et al. (2023)), the largest of which is the monophyletic Leptostemonum clade or the ‘spiny solanums’, characterised by the presence of prickles on the plant body (e.g. Satterlee et al. (2024)) and tapering, usually attenuate anthers.

The ’spiny solanums’ comprise approximately half the species diversity of the genus (ca. 600 species), but unlike the rest of *Solanum*, where species diversity is concentrated in the Americas, the clade has significant diversity in Africa, Asia, Australia (incl. New Guinea) and the Pacific (Bean 2004, 2016; McClelland 2012; Knapp and Vorontsova 2016; Vorontsova and Knapp 2016; Echeverría-Londoño et al. 2020; McClelland et al. 2020). Species outside the Americas largely fall into a single monophyletic ‘minor’ clade that has been called the Eastern Hemisphere Spiny clade (Gagnon et al. 2022), except for some members of the otherwise American Torva and Lasiocarpa clades (see Aubriot et al. (2016)). Group diversity in the Americas is more complex, with 18 ‘minor’ clades of spiny solanums occurring across the continent (Hilgenhof et al. 2023). Brazil is a hot spot of both species and clade diversity of spiny solanums; to date, 112 species in ten of the ‘minor’ clades of the Leptostemonum clade are recorded as native to Brazil and many taxa identified as new to science have also been identified and are awaiting description. Ongoing work on the Flora e Funga do Brasil (BFG 2021b) coupled with increased sampling of the many Brazilian species in molecular studies (e.g. Gouvêa (2020)), has revealed a number of morphologically coherent species groups within the spiny solanums that warrant monographic treatment.

**Figure 1.**
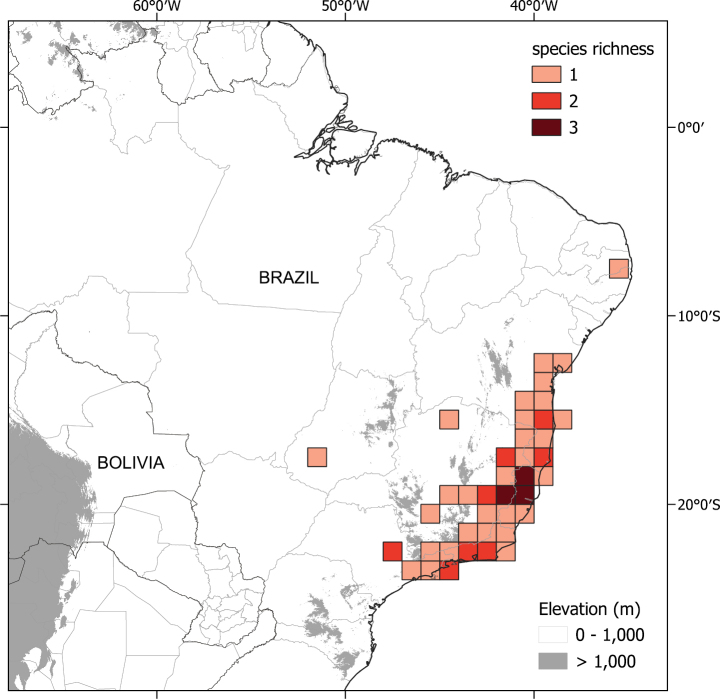
Map of distribution of the entire *S.hexandrum* group. Darker squares indicate higher species richness.

The six species treated here comprise one such small monophyletic group (see discussion of phylogeny below). All these species are endemic to the south-eastern Brazilian forests, with one species (S. *sublentum*) extending to central Brazil (Fig. 1, Table 1) and share strongly accrescent fruiting calyces, large repand leaves often with decurrent bases and large, robust flowers. We here treat this group as part of the wider effort to provide a monograph of *Solanum* at a global scale (Knapp et al. 2004) with detailed descriptions, keys and conservation assessments to aid understanding of Brazilian plant diversity and spur botanists to further collect and study the rare and fascinating species of this endemic group.

**Table 1.** Distribution of species of the *S.hexandrum* group. All species are endemic to Brazil.

Species	State(s)
*Solanumaciculare* Sw.	Bahia, Minas Gerais
*Solanumhexandrum* Vell.	Bahia, Espírito Santo, Minas Gerais, Rio de Janeiro, São Paulo
*Solanumhydroides* Gouvêa & Giacomin	Espírito Santo, Minas Gerais
*Solanumphrixothrix* Gouvêa & S.Knapp	Espírito Santo, [Minas Gerais?]
*Solanumstagnale* Moric.	Bahia, Minas Gerais, Paraíba
*Solanumsublentum* Hiern	Bahia, Espírito Santo, Goiás, Minas Gerais, Rio de Janeiro, São Paulo

### ﻿Taxonomy and relationships

Species of the *Solanumhexandrum* group were not described until the early 19^th^ century, despite their large, showy flowers. *Solanumaciculare* Sw. was the first species to be described in the group; Roemer and Schultes (1819) published a name and description they attributed to Olaf Swartz, based on a collection made by Georg Freyreiss who travelled with the prolific collector of Brazilian plants, Frederich Sellow (see description of *S.aciculare*). Roemer and Schultes (1819) had not seen the specimen itself, they only used Swartz’s name and description. *Solanumhexandrum* Vell., although possibly the earliest member of the group to be collected and recognised scientifically, was only published after *S.aciculare* in Brother José Mariano da Conceição Vellozo’s Flora Fluminensis (Vellozo 1829); Vellozo’s illustration (Vellozo 1831) is unambiguous and clearly shows the 6-parted corolla from which the epithet is derived. Vellozo (1829) also described *S.multiangulatum* Vell. that, from the illustration, was also possibly a member of this group, but the illustration and description are so rudimentary that the name is now suppressed (Knapp et al. 2015; See Names “designations” not validly published).

*Solanumstagnale* Moric. was described by Etienne Moricand (Moricand 1836) from the collections of the Swiss diplomat and botanist Jacques Samuel Blanchet. Blanchet was the Swiss consul resident in Bahia from 1826–1856 and collected many novelties in the region. *Solanumstagnale* was so named from the collection data stating “Hab. in stagnis circa Bahiam” (“habitat in [around] ponds near Bahia [probably Salvador]”). He did not contrast his new species with any other from Brazil.

In the treatment of *Solanum* in Flora Brasiliensis (Sendtner 1846), *S.aciculare*, *S.hexandrum* and *S.stagnale* were included in the large group of spiny solanums with straight prickles, lateral inflorescences and leaves that were not geminate, together with species with large, repand leaves now recognised as members of the Crinitum clade (*S.macranthum* Dunal = *S.crinitum* Lam.) and the Lasiocarpa clade (*S.sessiliflorum* Dunal) along with several other distinct clades of spiny solanums. Dunal (1852) included all three of the Brazilian species in his treatment of *Solanum* for the Prodromus (Dunal 1852), placing them in a group of spiny solanums distinguished by their more or less 5-parted corollas and more or less plicate corollas (“Corollis plus minusve 5-fidis, plus minusve plicatis” Dunal 1852: 30). He placed them in proximity with large-leaved species now considered part of the Crinitum clade, as had Sendtner (1846) before him.

*Solanumsublentum* Hiern was described some years later in a paper detailing species of *Solanum* occurring in central Brazil (Hiern 1878) described up to that time. It was compared to *S.sisymbriifolium* Lam., based on its prickliness and differing from it in shallowly lobed leaves, but not to the more closely-related *S.hexandrum* (treated by Hiern as *S.maroniense* Poit.).

Seithe (1962, 1979) developed a classification scheme for *Solanum*, based on hair types and included only *S.hexandrum* (as *S.maroniense*) in her system. She did not suggest affinities, but merely placed *S.hexandrum* as a member of her “subgenus Stellatipilum” (i.e. species with stellate trichomes). Danert (1970) followed Seithe’s sectional classification, although he based his descriptions on branching patterns rather than pubescence; he did not mention any of the species of the *S.hexandrum* group. D’Arcy (1972), in his lectotypification of the sections of *Solanum*, did not indicate where he thought these species belonged; he only indicated types for the subdivisions of *Solanum* and did not place any of these species in his system.

Whalen (1984) developed a species-group classification for the spiny solanums treating all species worldwide. He placed *S.hexandrum* and *S.stagnale* in his *S.polytrichum* species-group together with a miscellaneous set of taxa, based on their bristly pubescence and prickly accrescent calyces (*S.polytrichum* Moric., *S.hasslerianum* Chodat, *S.laniflorum* Sendtn. (= *S.polytrichum*), *S.rupincola* Mart. and *S.urticans* Dunal). These are all now considered more closely related to other species groups (Hilgenhof et al. 2023). Earlier (Whalen et al. 1981), he had treated *S.stagnale* as a member of section Lasiocarpa (Dunal) D’Arcy (= Lasiocarpa clade, called in Whalen (1984) the *S.quitoense* group) and in his conspectus (Whalen 1984) suggested it “bridges the gap in some respects between the *polytrichum* and *quitoense* groups” and that those two groups were probably closely related. He also suggested a relationship with his *S.erythrotrichum* group, based on decurrent leaves and reddish-brown pubescence (Whalen 1984: fig. 5).

Child (1998) formalised Whalen’s (1984) informally named group, typifying it with *S.polytrichum* (Child 1998; Child and Lester 2001). He excluded *S.urticans* from the group as he circumscribed it, but included *S.aciculare*; Child was the first to recognise *S.aciculare* as related to other species of the *S.hexandrum* group. Nee (1999) followed Whalen (1984), but excluded *S.hasslerianum* and *S.urticans* and added several poorly-known bristly Caribbean species (*S.gundlachii* Urb., *S.lomense* Britton & Wilson [= *S.gundlachii*], *S.schulzianum* Urb., *S.urens* Dunal). He suggested section Polytrichum A.Child as circumscribed by him “may not form a natural group” (Nee 1999: 317).

More recently and with increased interest and collecting associated with the Flora of Brazil project (BFG 2015, 2021a, 2021b), two taxa were described and associated with those three species that had been known and whose circumscription had remained essentially unchanged at the species level since the 19^th^ century. Remnant forests around granite inselbergs in Minas Gerais and Bahia states harboured *S.kollastrum* Gouvêa & Giacomin (now recognised as *S.aciculare*, see species treatment) and *S.hydroides* Gouvêa & Giacomin (Gouvêa et al. 2018, 2020).

Phylogenetic analysis of DNA sequence data has clarified monophyletic groups within *Solanum* (Levin et al. 2006; Gagnon et al. 2022) and strongly confirmed the monophyly of the large Leptostemonum clade, within which 19 minor clades were recognised. The highest lineage diversity in the spiny solanums is in the Americas (Särkinen et al. 2013); 18 of the 19 clades are from the Americas. All spiny species from the Eastern Hemisphere (i.e. Africa, Asia, Australia, Europe and the Pacific) grouped in one poorly-resolved clade (termed the EHS clade) comprising half of the spiny solanum diversity.

Only two species of the group (*S.hexandrum* and *S.stagnale*) were sampled in the first large scale molecular phylogeny of the spiny solanums (Stern et al. 2011); they resolved as sister and, together with *S.robustum* H.Wendl., formed a moderately supported clade sister to species previously recognised (Agra 2004, 2007) as section Erythrotrichum A.Child. Särkinen et al. (2013) and Gagnon et al. (2022) recovered the same relationship. These studies made clear that the previously postulated close affinities of *S.hexandrum* and *S.stagnale* to other spiny solanum species with large, repand leaves like *S.sessiliflorum* Dunal (Lasiocarpa clade) and *S.crinitum* Lam. (Crinitum clade) were more likely due to convergence in leaf shape than to evolutionary relationship.

A study employing full plastome sequences and focusing on Brazilian spiny solanums included all of the taxa recognised here, except *S.hydroides* and *S.phrixothrix* (i.e. *S.aciculare*, *S.hexandrum*, *S.stagnale* and *S.sublentum*) and further clarified monophyletic species groups in Brazilian spiny solanums (Gouvêa 2020). In these analyses, all sampled species formed a monophyletic group that was sister to a set of closely-related groups including species such as *S.jussiaei* Dunal, *S.rupincola* Sendtn., *S.cordifolium* Dunal and *S.oocarpum* Sendtn. *Solanumrobustum*, considered a member of the *S.hexandrum* group by Stern et al. (2011), resolved as sister to a group containing the poorly-known species *S.schizandrum* Sendtn. and an undescribed species (Gouvêa 2020). Together, the *S.hexandrum* group and these additional groups were sister to members of the Erythrotrichum clade s.s. and the Thomasiifolium clade, forming a group Gouvêa (2020) called “Erythrotrichum s.l.”. Based on these results, we have not included *S.robustum* in our treatment of the *S.hexandrum* group. As many of the species in these groups are undescribed and poorly characterised, it is clear that more studies are needed, employing both nuclear markers and morphology, to untangle these largely Brazilian groups of spiny solanums.

### ﻿Morphology

#### ﻿Habit

**Figure 2.**
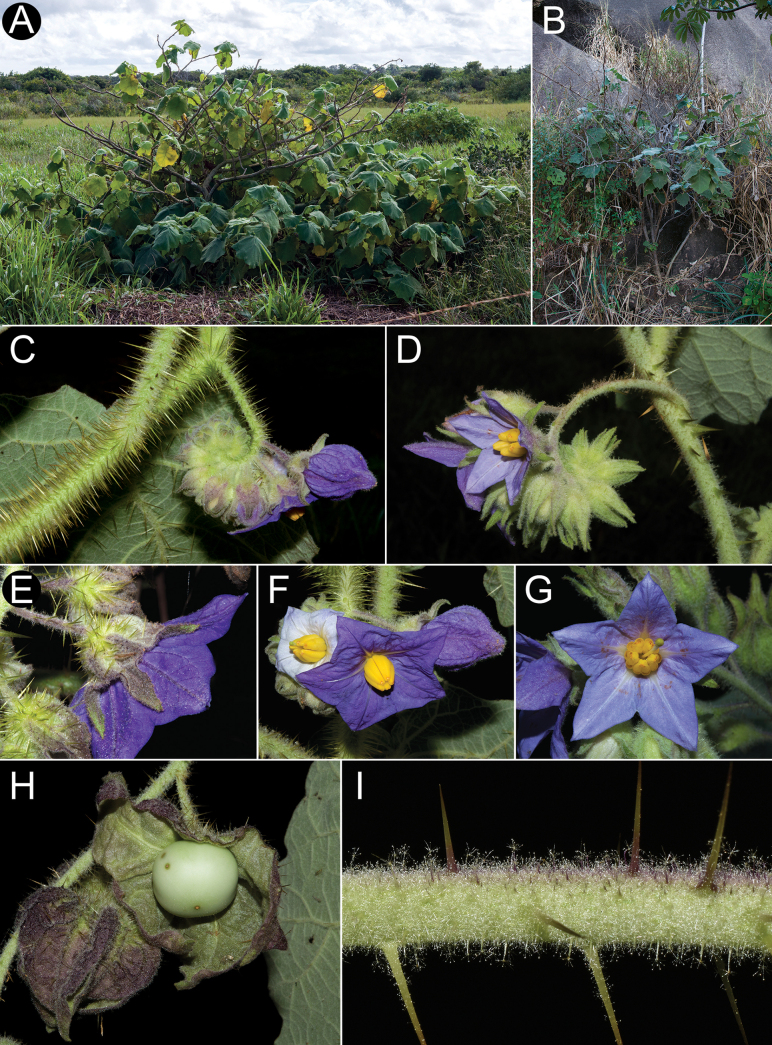
*Solanumaciculare***A** habit in open area **B** habit along rock face **C** inflorescence with bud **D** inflorescence with open short-styled flower **E** calyx at anthesis **F** short-styled flowers (note colour change over anthesis) **G** long-styled flower **H** mature berry with calyx removed **I** stem section with glandular long-stalked stellate and multangulate trichomes and straight acicular prickles (**A**, **D**, **G***Gouvêa 283*; **B**, **H***Gouvêa 281*; **C**, **E**, **F**, **I***Gouvêa 280*). Photos: Yuri F. Gouvêa .

Members of the *Solanumhexandrum* group are all medium- to large-sized shrubs. Amongst the largest plants are *Solanumaciculare* and *S.hexandrum*, both often reach 3 metres tall in shaded environments with considerably robust stems (to 2–3 cm diameter at base in some populations of *S.hexandrum*), that are nevertheless soft-wooded. The strongly-branched stems that are fully covered with very long, straight prickles in *Solanumaciculare* give it an extremely robust appearance, even when quite small (Fig. 2C). *Solanumstagnale* and some populations of *S.hexandrum* are shorter plants, reaching no more than 2–2.5 m. *Solanumhydroides* and *S.sublentum* are more delicate plants, with relatively slender stems. No rhizomatous growth is reported in the group.

#### ﻿Stems

The stems in the *Solanumhexandrum* group are organised as a typical *Solanum* sympodium, with a 2/5 phyllotaxic spiral with inflorescences emerging laterally (internodal) or subopposite to the leaves (Danert 1958). All species of the clade have monochasial branching (Danert 1958). Plants from shaded environments, like some populations of *S.hexandrum*, are commonly observed in flower with a single unbranched stem with robust and showy flowers easily seen from a considerable distance. The sympodial units are mostly difoliate and the leaves usually not geminate, but unifoliate sympodial units are occasional in *S.hexandrum* and *S.aciculare* and *S.phrixothrix* have plurifoliate sympodial units. The exact nature of the sympodial unit in these plants, however, can be difficult to see, as the plants are large and herbarium specimens often consist of very few leaves and a single inflorescence.

#### ﻿Leaves

All species of the *Solanumhexandrum* group have large, simple repand leaves that are elliptic to ovate in outline and usually lobed to some extent (see Figs 2, 4, 5, 7, 9, 11, 13). Most of these leaves are membranous, but look thicker due to the dense pubescence; in live plants, they are somewhat floppy and soft. It is important to observe that great variation in leaf size and width is observed between young and mature plants and when comparing plants from shaded and sunny environments in all species, with larger leaves reported in young individuals from shaded environments (Gouvêa et al. 2020). The greatest variation, nevertheless, is found in species that can be found associated with granitic outcrops (inselbergs), such as *S.hydroides* and *S.hexandrum* (especially the glabrous individuals of the latter), with much smaller leaves observed in specimens growing on the rocks of outcrops as compared to those from shaded forest edges. As leaves of members of the clade can be quite large, reaching 45 cm or more long, it is common to find herbarium specimens with leaf sizes that do not represent what is observed in nature. Stem apices are preferentially collected so that they fit in a standard herbarium sheet (ca. 45 × 30 cm) and because this is where flowers are fruits are borne.

Leaf bases can be decurrent on to the petiole in *S.hexandrum*, *S.hydroides* and *S.stagnale* (see Figs 4B, 11C), not or rarely decurrent in *S.sublentum* (Fig. 13F, G) or not at all decurrent (*S.aciculare*, *S.phrixothrix*). Leaf margins can be entire (e.g. *S.hexandrum*), shallowly to deeply lobed or lobed with few to many secondary lobes. Young individuals and resprouts of most species produce more deeply-lobed leaves, often with more abundant and prominent secondary lobing. As the plants develop, more distally produced leaves are more shallowly lobed, with sparser and usually less pronounced secondary lobing or with this secondary lobing completely absent. *Solanumsublentum* is the only species in the group whose leaves retain numerous secondary lobes in more developed and older individuals (Fig. 13F, G).

Leaf texture also varies greatly and is mostly related to the environment and type of trichome developed. Species growing in open environments, such as *S.hydroides* and some populations of *S.hexandrum* tend to have trichomes with multiseriate bases that can be rough to the touch, especially when growing on rocky outcrops or exposed forest clearings. The leaves of *S.sublentum* are quite soft to the touch, but sticky due to the presence of glandular trichomes. *Solanumaciculare* is strongly glandular pubescent (Fig. 2), but a single specimen (*Magalhães 17651*, IAN) appears to be less glandular pubescent than other collections we have seen; we have, however, only seen this as an image and the glandular nature of the stellate trichomes of *S.aciculare* is hard to see unless resolution is high.

#### ﻿Inflorescences

The inflorescences of members of the *Solanumhexandrum* group, as in most Solanaceae species, are developmentally terminal (Lippman et al. 2008), but they can appear lateral due to subsequent branch elongation from the subtending axillary bud. Most species in the group have unbranched inflorescences, but we have seen one specimen of *S.hexandrum* with a forked inflorescence, perhaps due to injury. Inflorescences of *S.hydroides* and *S.sublentum* usually have relatively short axes (Figs 7C, 13H) bearing only a few flowers and more robust axes than are found in *S.aciculare* and *S.hexandrum*. *Solanumphrixothrix* has a long, slender inflorescence axis with flowers borne only near the tip (Fig. 9A).

The number of flowers per inflorescence varies greatly amongst species of the clade, ranging from a few (3–6) in *S.sublentum* and *S.hydroides*, to many (up to 35) in *S.aciculare* and *S.phrixothrix*. There are commonly only one or two flowers open at a time, even in species like *S.sublentum* that is not markedly andromonoecious (see Breeding systems below).

#### ﻿Pubescence

Like all members of the Leptostemonum clade, members of the *S.hexandrum* group have stellate trichomes, composed of a stalk of varying lengths (including absent and the trichomes sessile), lateral rays and a central mid-point (see Roe (1971); Vorontsova and Knapp (2016)). Some of these hairs can appear simple, due to loss of the lateral rays, but are derived from stellate trichomes (Roe 1971; Nee 1979) and are here and in the descriptions described as stellate or modifications thereof. Pubescence in the group is variable, particularly in *S.hexandrum* (see below). Most species of the group have stalked trichomes, with multiseriate stalks composed of 2-many rows of cells. Stalks with more rows of cells are more robust and generally stiffer. Rays (lateral branches) are usually single-celled, porrect and all in a single plane (Fig. 7E), but are sometimes multangulate and pointing in different planes (see Fig. 2I). The mid-point is 1-2(3) cellular and is often shorter (e.g. *S.hexandrum*, *S.hydroides*) than the ray cells, but, in *S.aciculare*, the mid-point can be much longer than the rays. The trichomes of *S.aciculare*, *S.sublentum* and *S.stagnale* are gland-tipped; those of *S.aciculare* are unusual in *Solanum* in having glandular tips on not only mid-points. but also on all of the ray cells (Fig. 2I).

*Solanumphrixothrix* and *S.sublentum* have apparently simple uniseriate trichomes that appear to be modified stellate trichomes like those that occur in the Acanthophora clade (Nee 1979; Hilgenhof et al. 2023), in *S.sublentum*, these are accompanied by a subtending layer of sessile rayed stellate trichomes and, in the few specimens seen, all trichomes of *S.phrixothrix* lack rays (Fig. 9B). Some populations of *S.hexandrum* appear to be completely glabrous, lacking trichomes altogether. In others, plants are pubescent, but with apparently simple trichomes like those of *S.phrixothrix*, with the rays lost such that the multiseriate stalk appears to be an unbranched multiseriate trichome topped with a 1-celled mid-point, this is often bent at right angles to the stalk making the trichome a distinctive L-shape (Fig. 5L). These are often characterised as bristles. Pubescence density in *S.hexandrum* is extremely variable, but the distinctive bent trichomes are consistent across the species range.

Most species of this group have dense pubescence on all parts, except for some populations of *S.hexandrum* (see species description and discussion). In general, pubescence of abaxial leaf surfaces is denser than that of adaxial surfaces; in *S.stagnale*, the leaf lamina is not visible on either surface due to the dense covering of long-stalked trichomes subtended with a lower layer of sessile stellate trichomes. Field observations of several species indicate that trichomes can be purple-tinged (e.g. *S.aciculare*, *S.hexandrum* and *S.hydroides*) and those of *S.stagnale* are usually characterised as reddish-brown or red, but can also be somewhat purple-tinged in live plants (Fig. 11C).

#### ﻿Prickles

In *Solanum*, prickles are considered to be modified trichomes (Whalen 1984) as can be seen by them often having apical stellae (Vorontsova and Knapp 2016). They have been lost in several groups and species; all losses are due to modifications in a single gene (Satterlee et al. 2024). There is a fine gradation between bristles and prickles (Vorontsova and Knapp 2016; see descriptions of *S.setaceum* Dammer and *S.schumannianum* Dammer), with less sturdy structures being termed bristles (e.g. in this group *S.phrixothrix*, Fig. 9B) and more robust structures called prickles. In species of the *S.hexandrum* group, prickles can be distinguished from bristle-like trichomes by their pointed tips, which lack rays and mid-points. Bristle-like trichomes, on other hand, always have a unicellular or 2–3-celled uniseriate mid-point of varying lengths, which leaves a scar when deciduous.

In the *S.hexandrum* group, prickles occur throughout the plant, but are most prominent on stems and along the leaf venation. Prickles also occur on inflorescence axes and calyces in most species, but are generally smaller than those of stems. Prickles in the spiny solanums are either needle-like (acicular) or laterally flattened (Hilgenhof et al. 2023); in this group, stem prickles are usually laterally compressed and widest at the base, although *S.phrixothrix* has acicular bristles that are not markedly flattened (Fig. 9B). Straight stem prickles are found in *S.aciculare*, *S.hexandrum* and *S.phrixothrix*, but those of *S.hydroides*, *S.stagnale* and *S.sublentum* are moderately to strongly curved (Figs 2I, 5B, 7C, 11E, 13E).

Prickle density and size varies both between and within species. Most stem prickles in this group are between 0.5 and 1 cm long, whereas prickles along leaf venation are usually shorter. *Solanumaciculare* and *S.phrixothrix* are consistently densely prickly/bristly, whereas *S.stagnale* is quite variable. Some individuals of *S.stagnale* have large, densely-spaced recurved prickles along the stems.

#### ﻿Pedicels

Flower and fruit pedicels in the *S.hexandrum* group are usually quite robust and vary from being almost absent (*S.stagnale*, Fig. 11C, F) to 2 cm long (e.g. *S.hexandrum*, Fig. 5B). *Solanumhydroides* and *S.phrixothrix* have the most delicate pedicels in the group (Figs 7C, 9C). They usually have at least some prickles, especially in the lower flowers that are co-sexual (see below and species descriptions). Pedicels are usually spreading to erect at anthesis, whereas in fruit, they are usually somewhat deflexed from the weight of the large berries (Fig. 5J).

#### ﻿Calyx

Calyces in members of the *S.hexandrum* group are typically sympetalous and 5-merous as most species of *Solanum* (although as the specific epithet implies that those of *S.hexandrum* are often 6-merous). The shape of the sepals and how they develop in fruit can be informative for species recognition. In *S.hydroides* and *S.hexandrum*, the sepals are generally deltate to lanceolate (Figs 4, 5, 7), they are somewhat tongue-shaped to spathulate in *S.stagnale* (Fig. 11C) and expanded at the base and somewhat cordate in *S.aciculare* (Fig. 2E) and *S.sublentum* (Fig. 13G). The calyx in fruit is usually to some degree inflated and accrescent, often covering half or more of the mature berry, only in *S.hydroides*, *S.stagnale* and some populations of *S.hexandrum* does the calyx remain closely appressed to the developing berry (Figs 4J, 7E, 11F). In *S.aciculare*, *S.sublentum* and some populations of *S.hexandrum*, the calyx in both flower and fruit is strongly inflated (Figs 5J, 13J) and the base is invaginate. In *S.hexandrum*, this accrescent condition can vary (e.g. Fig. 4J) and is often not visible in flower. In some populations of *S.hexandrum*, the calyx appears to be fused throughout development, opening only just before anthesis (Fig. 5C–E); this state occurs more often in glabrous or sparsely pubescent individuals.

Calyces of all these species are often dark purple or purple-tinged in fruiting plants. Ants of the genus *Camponotus* have been seen foraging on exudate from the abaxial surface of the calyx of *S.hexandrum* (YFG, pers. obs.); this has only observed in the glabrous to sparsely hairy plants, but may be more common as has been seen in other groups of spiny solanums (e.g. Lasiocarpa clade, Anderson and Symon (1985); Falcão et al. (2003)).

#### ﻿Corolla

The corolla in members of the *S.hexandrum* group is sympetalous and 5(6)-merous and relatively large compared to most other members of the Leptostemonum clade. The smallest corollas are found in *S.hydroides* (2.4–3 cm in diameter) and the largest in *S hexandrum* (3–6 cm in diameter). Most species have somewhat stellate corollas, lobed up to halfway to the base, with deltate to broadly triangular lobes. *Solanumphrixothrix* has a rotate to rotate-pentagonal corolla with only very tiny, minutely apiculate lobes (Fig. 9C, E, F).

Corollas in these species are usually various shades of purple, fading to paler shades with age. In *S.aciculare*, the corollas are dark purple at the onset of anthesis (Fig. 2F, G), becoming white when old. *Solanumsublentum* is polymorphic, with individuals with white corollas (Fig. 13H inset) and others with purple (Fig. 13G, H). *Solanumhydroides* and *S.phrixothrix* have uniformly white corollas.

Where abaxial petal tissue is exposed in bud, it is usually pubescent with stellate trichomes like those of the rest of the inflorescence (Fig. 4G); interpetalar tissue, however, is always thin and glabrous. Adaxial petal surfaces are usually glabrous, but sparse stellate trichomes (e.g. *S.aciculare*) and sometimes minute straight prickles (e.g. *S.sublentum*) occur along the petal mid-veins and apices. The tips of the corolla lobes are often somewhat apiculate (Fig. 2G, uppermost corolla lobe).

#### ﻿Androecium

As in all species of *Solanum*, anthers are borne on short filaments and are poricidal at the tips. In all species of the *S.hexandrum* group, the androecium is monomorphic, with stamens and anthers are of equal size (e.g. Fig. 2F). Anthers are loosely connivent and colour is uniformly recorded as yellow on herbarium labels. The anthers of members of the group are somewhat plump in the lower half and not as attenuate as in other groups with large flowers (e.g. *S.crinitum* or *S.lycocarpon* A.St.-Hil., both of the Crinitum clade), but in common with other members of the Leptostemonum clade, they are narrowed apically. The abaxial surfaces are swollen (making the anthers somewhat gibbous) and sometimes papillate, but this is not constant within a species. The apical pores do not lengthen to slits with age or drying and are usually directed distally and somewhat extrorsely.

#### ﻿Gynoecium

Тhe ovary in members of this group is 2–4-locular with axile placentation. The ovary is usually somewhat pubescent apically with a tuft of stellate trichomes, these being deciduous or mostly deciduous throughout fruit development. The style is cylindrical, straight and glabrous; in long-styled flowers, it is exserted from the anther tube, whereas in short-styled flowers (usually distal on the inflorescence), it is held well-within the anther tube (see Fig. 9D). The stigma is usually large, clavate or globose-capitate and is occasionally bilobed; stigmas in live plants are bright green (see Figs 2G, 7D, 11E, 13I).

#### ﻿Fruit

The fruit of all members of the *S.hexandrum* group is a 2–4-locular berry, usually enclosed in the accrescent calyx (Fig. 7F). Mature berries of most species are completely enclosed in the accrescent calyx, but those of *S.hydroides* and *S.stagnale* are only partially enclosed with approximately half of the mature berry exposed (Figs 7E, 11F). The berries of these species are green, greenish-white or white at maturity with a fleshy pale white mesocarp and many small seeds (> 100 per berry); medium-sized greenish-white berries have been shown to be ancestral in *Solanum* (Messeder et al. 2024).

A single collection of *S.hexandrum* (*Magnago 467*, MBML) has been recorded as having a purple fruit, but it is probable that this observation refers to the accrescent calyx and not the berry itself (the specimen is in early fruit with completely accrescent calyces). A mild sweet scent has been recorded for *S.aciculare* (Gouvêa et al. 2018), but scent has not been recorded for any other species. Fruits are not known for *S.phrixothrix*.

#### ﻿Seeds

The seeds of these species are dark brown, relatively small within the Leptostemonum clade (ca. 2–2.6 mm long) and flattened reniform or somewhat ovoid with minutely-pitted testal cells (Fig. 7F). The testal cells are usually pentagonal or only slightly sinuate in outline, but *S.aciculare* and *S.sublentum* have testal cells that are markedly sinuate in outline.

#### ﻿Chromosomes

Chromosome numbers in *Solanum* are based on a base number of x = 12 (Chiarini et al. 2018). *Solanumstagnale* is the only one of the species treated that has a chromosome count. Bernardello et al. (1994) recorded a sporophytic count of 2n = 24 for *S.stagnale* (treated as a member of section Lasiocarpa) and showed that, morphologically, its karyotype was similar to that of *S.quitoense* (Lasiocarpa clade) with a formula of 10 m + 2 sm chromosomes.

### ﻿Ecology and natural history

#### ﻿Habitats

All of these species are endemic to Brazil (Fig. 1, Table 1). Members of the *Solanumhexandrum* group are associated with a wide range of habitats, from wet shaded forests to outcrops, mostly restricted to the Atlantic Forest domain in eastern Brazil, from the States of São Paulo to Paraíba. *Solanumsublentum*, however, also occurs in patches of seasonally dry forest (SDTF of Murphy and Lugo (1986); Pennington et al. (2009)) within a savannah matrix in the Cerrado domain at the limits of Cerrado vegetation in central and south-eastern Brazil in the States of Minas Gerais and Goiás (see Table 1 for the distribution of all species). Species are recorded from evergreen and seasonally semi-deciduous wet forests, in clay soils, to more open sandy coastal lowland vegetation (restinga, see Araújo (1992)) or associated with forest patches at the base of gneiss or granite inselbergs, where sandy or leached shallow soils are more common. *Solanumsublentum* is also found growing in soils with high levels of calcium and magnesium associated with limestone outcrops. but is not exclusive to this soil type.

*Solanumhexandrum* is the most widely distributed species ranging from São Paulo to southern Bahia State and has been collected both in and at the edges of evergreen and seasonal forests. *Solanumaciculare* and *Solanumhydroides* are restricted to gneissic/granitic outcrops together with some populations of *S.hexandrum*, but *S.aciculare* can also be found in disturbed restinga vegetation. *Solanumphrixothrix* is found in wet evergreen and semi-deciduous forests in the Rio Doce drainage, sometimes associated with forests on sandy soils known as “tabuleiro” (tableland) formations (Peixoto et al. 2008). All species have also been collected in anthropogenically disturbed habitats, by roads or in forest clearings.

#### ﻿Sex expression and breeding systems

Like many other members of the Leptostemonum clade, some species of the *S.hexandrum* group exhibit andromonoecy where the first (proximal) “long-styled” flower(s) have styles that protrude beyond the anthers (e.g. Figs 4G, 13I) and go on to develop fruits and the later (distal) “short-styled” flowers have partly developed styles and do not normally develop fruits (Fig. 2D, F)). Andromonoecy has been extensively studied in *Solanum* (e.g. Dulberger et al. (1951); Whalen and Costich (1986); Anderson and Symon (1989); Miller and Diggle (2003); Diggle and Miller (2013)) and is believed to be an adaptation to limited resources which allows the plant to restrict the number of costly fruits without decreasing pollen production. In the spiny solanums, there is a continuum from weakly andromonoecious species with a low proportion of staminate flowers (i.e. many co-sexual flowers, for example, *S.sublentum*) to strongly andromonoecious species with a high proportion of staminate flowers and only one hermaphroditic flower that sets fruit. A statistically significant correlation has been found between the strength of andromonoecy, larger fruit size, larger ovary size and larger size of long-styled flowers (Miller and Diggle 2003). Our phylogenetic studies across *Solanum* indicate that andromonoecy does not define monophyletic groups (Vorontsova et al. 2013; Aubriot et al. 2016). Breeding systems in the *S.hexandrum* group have not been studied in the field or laboratory and are postulated here, based on morphology.

#### ﻿Pollination and dispersal

Members of the *S.hexandrum* group have been the subject of little ecological study in the field. Based on the poricidal dehiscence and anther robustness, the main pollinators of these species are likely to be medium- to large-sized bees that gather pollen by buzzing the anthers (Bowers 1975; Buchmann et al. 1977; Vallejo-Marín 2019, 2022). A study of pollination networks in the forests around the inselberg of Itaoca (Rio de Janeiro State) recorded visits of bees from the families Apidae [*Bombusmorio* (Swederus), *Eulaemacingualata* (Fabricius), *Oxaeaflavescens* Klug), Halictidae (*Augochloropsis* sp., *Pseudaugochloropsisgraminea* (Fabricius)] and Megachilidae (*Megachile* sp.) to plants of *S.hexandrum* (Marques et al. 2018). It is likely that only the larger of these bees were effective pollinators. Species of *Bombus*, for example, are effective pollinators of *Solanum* in other habitats (Knapp 1986; Messinger et al. 2016). Smaller bees such as *Augochloropsis* often glean pollen from anther pores and may be less important in pollination of these large-flowered species (Whalen 1979; Solís-Montero and Vallejo-Marín 2017; Vallejo-Marín 2019).

Berries of members of the *S.hexandrum* group are variable in size (from 1–3.5 cm in diameter); they usually hang below the foliage on short or long inflorescence axes and are enclosed in an accrescent calyx. This combination of characteristics, coupled with the white or greenish-white colour at maturity and sweet smell (observed in *S.aciculare*) suggest dispersal by bats (Van der Pijl 1972; Valenta and Nevo 2020). Messeder et al. (2024) suggest that correlation of the emergence of medium-sized green fruits and the most diverse family of fruit-eating bats (Phyllostomatidae) is evidence for bat dispersal being ancestral in the genus. In the Brazilian Atlantic Forest, *Solanum* species have been shown to be important components of bat diets (Aguiar and Marinho Filho 2007; Mello et al. 2008) and the role of bats as dispersal agents for some *Solanum* species is well-documented (Iudica and Bonaccorso 1997; Paulino-Neto et al. 2014).

#### ﻿Conservation status

Most species of the *Solanumhexandrum* group are found in small populations with individuals growing close to each other, often in isolated forest patches at the base of granitic or other rocky outcrops. No vegetative reproduction has been reported for any species of the group. Although some species were previously informally assessed as threatened (*S.aciculare* as EN, Gouvêa et al. (2018); *S.hydroides* as VU, Gouvêa et al. (2020)), we assess all species of the group here for the first time (see Table 2 and species descriptions). Using AOO (Area of Occupancy), all of these species are identified as Endangered (see species treatments), but EOO (Extent of Occurrence) estimates suggest only *S.aciculare* and *S.hydroides* are of significant conservation concern (*S.phrixothrix* is Data Deficient due to scarcity of collections). All these species are probably subject to population size fluctuations or area of occupancy reductions due to anthropogenic landscape change. Urban centre expansion and conversion of native vegetation to alternative land uses, such as pastures or *Pinus/Eucalyptus* plantations for cellulose production (Ribeiro et al. 2009), are resulting in rapid fragmentation of the Atlantic Forest (Tabarelli et al. 2004). Half of the species treated here (e.g. *S.aciculare*, *S.hydroides* and *S.phrixothrix*) are found in the central Brazilian Atlantic Forest, where botanical knowledge gaps are known to exist (Stehmann et al. 2009; Oliveira et al. 2016). Rock outcrops in these regions harbour the last remnants of forest fragments (Martinelli 2007) and every remnant of native vegetation of rocky outcrops, no matter the size, is worth preserving and should be inspected. Except for *Solanumhexandrum*, most of these species have not been recorded from any or only a single protected area (see Preliminary Conservation Assessments for each species), suggesting they are all vulnerable to some degree.

**Table 2.** Preliminary conservation status of members of the *S.hexandrum* group.

Species	EOO (km^2^)	AOO (km^2^)	Preliminary status
*Solanumaciculare* Sw.	38, 227 (NT)	56 (EN)	Endangered
*Solanumhexandrum* Vell.	327,280 (LC)	460 (EN)	Near Threatened
*Solanumhydroides* Gouvêa & Giacomin	12,549 (VU)	24 (EN)	Endangered
*Solanumphrixothrix* Gouvêa & S.Knapp	--	--	Data Deficient
*Solanumstagnale* Moric.	157,059 (LC)	68 (EN)	Vulnerable
*Solanumsublentum* Hiern	642,872 (LC)	72 (EN)	Near Threatened

## ﻿Materials and methods

Our taxonomic treatment is based on study of herbarium specimens and plants in the field. Delimitation and descriptions are based on fieldwork and examination (physical and virtual) of 749 [= 460 collections] herbarium specimens from 64 herbaria: A, ALCB, BH, BHCB, BM, BR, C, CEPEC, CESJ, CORD, CVRD, E, EFC, ESA, F, FUEL, FURB, G, G-DC, GH, GOET, HAS, HCF, HRCB, HSTM, HUEFS, IAC, IAN, IBGE, ICN, JPB, K, L, LE, M, MA, MBM, MBML, MCCA, MG, MO, NIT, NY, OXF, P, RB, RFA, RFFP, S, SI, SP, SPF, SPSF, TCD, U, UCPB, UEC, UNOP, UPCB, US, UT, VIC, W and WU. Some of these specimens were examined digitally through individual herbarium portals; we include only those specimens which we have been able to unequivocally identify from these images or that are duplicates of collections we have personally examined.

Measurements were made from dried herbarium material supplemented by measurements and observations from living material. Colours of vegetative organs (e.g. leaves, prickles and trichomes) and seeds are described from dried herbarium collections (and living plants when available) and for corollas, fruits etc., are described from living material or from herbarium label data. Specimens with latitude and longitude data on the labels were mapped directly. Most species had few or no georeferenced collections; here, we retrospectively georeferenced the collections using available locality data. Maps were constructed with the points in the centres of degree squares in a 1° square grid. Conservation threat status was assessed following the guidelines for the IUCN Red List Categories and Criteria (IUCN 2020) using the GIS-based method of Moat (2007) as implemented in the online assessment tools in GeoCat (http://geocat.kew.org; Bachman et al. (2007)). The Extent of Occurrence (EOO) measures the range of the species and the Area of Occupancy (AOO) represents the number of occupied points within that range, based on the default grid size of 2 km^2^. We have taken a pragmatic approach in the threat assessments for most species, especially where assessments based on EOO and AOO differ widely; AOO is very sensitive to georeferencing bias and collecting effort, but the extreme vulnerability of these habitats is clear.

Where specific herbaria have not been cited in protologues, we have followed McNeill (2014) and designated lectotypes rather than assuming holotypes exist. We cite page numbers for all previous lectotypifications. In general, we have lectotypified names with the best preserved or, in some cases, with the only herbarium sheet we have seen; in these cases, we have not outlined our reasoning for the lectotypifications. Where there has been difficulty or where the choice may not be obvious, we detail our reasoning at the end of the species discussions. For names that have been “inadvertently” lectotypified (*sensu*Prado et al. (2015)), we indicated what the original author cited (e.g. “type”, “holotype”) after the lectotype citation.

Type specimens with sheet numbers are cited with the herbarium acronym followed by the sheet number (e.g. SD [acc. # 6543]); barcodes are written as a continuous string in the way they are read by barcode readers (e.g. G00104280, MO-1781232). For those herbaria (e.g. A, GH, NY, US) where the barcode consists of only a number, we cite only the number string. Where herbaria have both barcodes and accession numbers, we always cite the barcode first, followed by the accession number (e.g. MO-503846, acc. # 3783069); this citation will allow users to access individual sheets where barcode numbers are not human-readable.

Identities of all collections seen for this study are presented in Suppl. materials 1, 2 with full searchable specimen details available in Suppl. material 1 and all collection events in Suppl. material 2. All these files of specimens used for this study are also deposited in the Natural History Museum Data Portal (https://doi.org/10.5519/vv8f8pkx).

Citation of literature follows BPH-2 (Bridson 2004) with alterations implemented in IPNI (International Plant Names Index, http://www.ipni.org) and Harvard University Index of Botanical Publications (http://kiki.huh.harvard.edu/databases/publication_index.html). Following Knapp (2013), we have used the square bracket convention for publications in which a species is described by one author in a publication edited or compiled by another (the traditional “in” attributions), as, for example, Dunal in DC. for those taxa described by Dunal in Candolle’s *Prodromus Systematis Naturalis Regni Vegetabilis*. This work is cited here as Prodr. [A.P. de Candolle] and the names are thus attributed only to Dunal. For “ex” attributions, we cite only the publishing author, as suggested in the *Code* (Turland et al. 2018). Standard forms of author names are according to IPNI (International Plant Names Index, http://www.ipni.org).

### ﻿Species concepts

Our goal for this treatment has been to provide circumscriptions for the members of this morphologically variable group of species, while also clearly highlighting areas, taxa and populations where further in-depth research would be useful. Delimitation of species here basically follows what is known as the “morphological cluster” species concept (Mallet 1995; Knapp 2008): i.e. “assemblages of individuals with morphological features in common and separate from other such assemblages by correlated morphological discontinuities in a number of features” (Davis and Heywood 1963). Biological (Mayr 1982), phylogenetic (Cracraft 1989) and the host of other finely-defined species concepts (see Mallet (1995)) are almost impossible to apply in practice and are, therefore, of little utility in a practical sense (see Knapp (2008)). It is important, however, to clearly state the criteria for the delimitation of species, rather than dogmatically follow particular ideological lines (see Luckow (1995); Davis (1997)). We have relied on clear and consistent morphological discontinuities to define species. Specific characters used for recognition are detailed with each species description and in the key. In this revision, we have tried to emphasise similarities between populations instead of differences, which so often reflect incomplete collecting or local variation. We have been conservative in our approach, recognising as distinct entities those population systems (sets of specimens) that differ in several morphological characteristics. We describe and illustrate variation occurring within more variable species (e.g. *S.hexandrum*) realising that others may wish to interpret it differently.

## ﻿Taxonomic treatment

**The *Solanumhexandrum* group**

*Solanumhexandrum* clade, sensu Stern et al. (2011) pro parte.

SolanumsubsectionAsterotrichotum Dunal, Prodr. [A.P. de Candolle] 13(1): 30. 1852, pro parte.

*Solanumpolytrichum* species group of Whalen, Gentes Herb. 12: 248. 1984, pro parte (excl. *S.hasslerianum* Chodat, *S.polytrichum* Moric., *S.urticans* Dunal).

*Solanumquitoense* species group of Whalen, Gentes Herb. 12: 249. 1984, pro parte.

SolanumsectionPolytrichum (Whalen) A.Child, Feddes Repert. 109: 422. 1998, pro parte (excl. type species *S.polytrichum*).

**Description.** Shrubs, erect, sparsely to densely armed. Stems terete, glabrous to densely pubescent and/or bristly, usually prickly; trichomes porrect-stellate or multangulate, eglandular or glandular, sessile to long-stalked, the stalks multiseriate and usually robust, the rays (2)-4-20, often deciduous or missing and the trichomes appearing simple; prickles straight or recurved, acicular or laterally compressed, often widest at the base and with scattered stellate trichomes on the prickle itself. Sympodial units unifoliate, difoliate or plurifoliate, the leaves not geminate. Leaves simple, usually shallowly lobed, repand; blades glabrous to densely stellate-pubescent, usually prickly, the trichomes eglandular or glandular, porrect or multangulate, sometimes the stellate trichomes without rays and appearing simple, the prickles generally along the mid-rib and veins, smaller than those of the stems; base attenuate to truncate or cordate, often decurrent on to the stem; margins usually shallowly lobed, sometimes secondarily so, the basiscopic lobes, if present, rounded to angular; apex acute to acuminate; petioles winged or not. Inflorescences internodal or subopposite the leaves, unbranched (a single specimen of *S.hexandrum* seen with a furcate inflorescence), with 3–30 or more flowers, usually only a few open at a time, glabrous to densely stellate-pubescent and/or bristly, often prickly; peduncle usually elongate; pedicels very short to ca. 2 cm long, glabrous to stellate-pubescent and/or bristly, articulated at the base, the trichomes glandular or eglandular. Flowers 5-merous (sometimes 6-merous in *S.hexandrum*), actinomorphic, heterostylous, co-sexual (long-styled) flowers either along the entire inflorescence or borne near the base, staminate (short-styled) flowers borne distally, the plants varyingly andromonoecious; calyx with the tube often enlarged and saccate, sometimes plicate at the junction of the lobes, glabrous or more usually variously stellate-pubescent, prickly or bristly, the lobes narrowly to broadly triangular to spathulate, often foliose, usually enclosing the bud until just before anthesis, sometimes completely fused until just before anthesis; corolla large, usually purple, but, in some species, white, stellate to rotate, the lobes usually planar at anthesis, but, in *S.phrixothrix*, somewhat campanulate, interpetalar tissue absent to copious; stamens 5 (or sometimes 6 in *S.hexandrum*); filaments equal, glabrous; anthers equal, plump and tapering, connivent, abaxially often papillate and swollen in the lower half, dehiscing by apical pores; ovary 2–4-locular, glabrous or with a few apical glandular or eglandular stellate trichomes; styles straight or slightly curved, usually white, glabrous, in short-styled flowers held within the anther tube. Fruit a berry, usually 4-locular, up to 3.5 cm in diameter, often somewhat compressed, pale green to white at maturity, the mesocarp fleshy, the pericarp glabrous or with a few scattered stellate trichomes near the apex; fruiting pedicels usually deflexed from the weight of the berry; fruiting calyx variously accrescent, partially or completely enclosing the berry, appressed or saccate and invaginate; seeds many per berry, flattened reniform or somewhat ovoid, the surfaces minutely pitted, the lateral testal cell walls straight or sinuate; stone cells always absent. Chromosome number: n = 12 (Bernardello et al. 1994).

**Distribution and ecology.** Members of the *S.hexandrum* group are mostly endemic to eastern Brazil, ranging from São Paulo State in the south to Paraíba State as the northern limit, with a single species, *S.sublentum*, extending to the State of Goiás in central region of the country (see Fig. 1). All species occur in the Mata Atlântica, or Atlantic Rainforest, in a variety of environments, from shaded forest environments in clay soils to open exposed environments in sandy soils, often in association with gneissic/granitic inselbergs.

**Discussion.** This small group of species, all endemic to Brazil, is morphologically distinctive in having large, repand leaves, usually copious bristly pubescence on all parts, large flowers and accrescent fruiting calyces enclosing pale green to white mature berries. There is considerable morphological variation in some species (*S.hexandrum*) that needs further study. Most of these species have only rarely been collected, are found in small populations mostly outside of protected areas and are likely to be of conservation concern.

The spiny, shrubby Brazilian species of the Crinitum clade (e.g. *S.crinitum* Lam., *S.falciforme* Farruggia and *S.medusae* Gouvêa; Gouvêa et al. (2019)) can also have large, showy purple flowers, large leaves and bristly pubescence. However, species of the *S.hexandrum* group are easily distinguishable from these by their robust, abaxially glabrous anthers (rather than slender, stellate-pubescent anthers) and medium-sized fruits up to 3.5 cm in diameter enclosed or partially covered by accrescent calyces (as opposed to large fruits over 5 cm in diameter that are not covered by the calyces).

Other somewhat robust, large-leaved, spiny species occur within the range of the *S.hexandrum* group and can be superficially confused with them (e.g. *S.asterophorum* Mart., Asterophorum clade; *S.robustum* H.Wendl., Erythrotrichum clade). Although species of both the Asterophorum clade and the *S.hexandrum* group have accrescent fruiting calyces, they can be easily distinguished by their leaves, which are geminate (paired at the same node) in species of the Asterophorum clade, but not geminate in species of the *S.hexandrum* group. The leaves of *S.robustum* are similarly decurrent to those of some species of the *S.hexandrum* group, but in that species, the fruiting calyces are not accrescent and berries are densely pubescent at maturity, whereas in members of the *S.hexandrum* group, fruiting calyces are markedly accrescent and berries are not densely pubescent at maturity.

### ﻿Artificial key to the species of the *S.hexandrum* group

**Table:** 

1	Leaf bases cordate to hastate or sagittate-hastate with angular to rounded basiscopic lobes, not decurrent onto the petiole, rarely with a short and narrow attenuate extension < 1/2 the length of the petiole; leaf margins usually with secondary lobing	**2**
–	Leaf bases rounded, obtuse, attenuate or truncate, strongly decurrent onto all or most of the petiole; leaf margins without secondary lobing	**4**
2	Pubescence mostly glandular of stellate or a mixture of simple and stellate trichomes; calyx tube plicate from expanded lobe bases, especially in fruit; corollas shallowly to deeply stellate with deltate lobes	**3**
–	Pubescence eglandular of long, bristle-like trichomes; calyx tube not plicate; corollas rotate-apiculate	***Solanumphrixothrix***
3	Stem and petiole trichomes strictly stellate, long-stalked and multiglandular; corolla stellate, lobed to half of its length; leaf bases rounded-cordate, with basal-most lobes touching or overlapping each other over the petiole; leaf lobe apices rounded to obtuse in fully developed leaves of adult plants	***Solanumaciculare***
–	Stem and petiole trichomes mostly or strictly without rays and uniseriate, any stellate trichomes (when present) eglandular, sessile to short-stalked and less abundant; corolla pentagonal to very shallowly stellate, lobed to one quarter of its length; leaf bases cordate-angular to hastate or sagittate-hastate, with basal-most lobes never touching or overlapping each other over the petiole; leaf lobe apices acute with straight or concave margins in fully developed leaves of adult plants	***Solanumsublentum***
4	Mature leaves with both surfaces glabrous or very sparsely pubescent adaxially, the stellate trichomes lacking rays, bristle-like and bending; stems glabrous or with scattered stellate trichomes; calyx lobes in bud fused, enclosing the corolla almost to anthesis; calyces glabrous or with scattered trichomes, prickles and/or bristles	***Solanumhexandrum***
–	Mature leaves with both surfaces pubescent, the trichomes stellate or robust and bristle-like on the adaxial surface; stems pubescent with stellate trichomes or with a mix of stellate and unbranched bristle-like trichomes; calyx lobes in bud splitting before anthesis; calyces moderately to densely pubescent, also with prickles and/or bristles	**5**
5	Pedicels at anthesis up to 0.5 cm long, usually shorter; leaves and stems densely pubescent with multi-rayed trichomes; calyx lobes spathulate	***Solanumstagnale***
–	Pedicels at anthesis > 0.5 mm long, usually 1–2 cm long (if < 0.5 cm long, then the flowers white); leaves and stems glabrous to pubescent or bristly, at least some trichomes, when present, few-rayed, or bristle-like and unbranched; calyx lobes triangular to deltate	**6**
6	Corollas purple, 3–6 cm in diameter, often 6-merous; plants robust, usually > 1 m tall; blades of fully developed leaves > 13 cm long in adult plants; fruiting calyx accrescent, completely to almost completely covering the berry, inflated or loosely investing the berry, the lobes overlapping; berry 2–3.5 cm in diameter	***Solanumhexandrum***
–	Corollas white, 2.4–3 cm in diameter; plants delicate, usually < 1.5 m tall; blades of fully developed leaves ≤ 13 cm long in adult plants; fruiting calyx partially accresent, not completely covering the berry, tightly appressed, the lobes not overlapping; berry 0.9–1.8 cm in diameter	***Solanumhydroides***

### ﻿Synoptic character list for members of the *S.hexandrum* group

Leaf bases decurrent on to petiole and stem: *hexandrum*, *stagnale*, *hydroides*.

Leaf bases cordate: *aciculare*, *sublentum*, *phrixothrix*.

Stellate trichomes apparently absent: *hexandrum*, *phrixothrix*.

Trichomes glandular: *aciculare*, *sublentum*.

Trichomes unbranched (modified stellate trichomes without rays): *hexandrum*, *phrixothrix*, *sublentum*.

Prickles broad-based: *hexandrum*, *stagnale*.

Corolla rotate to pentagonal: *phrixothrix*.

Fruiting calyx invaginate (plicate, sometimes only obvious on immature fruits): *aciculare*, *sublentum*.

### ﻿Species descriptions

#### ﻿1. Solanum aciculare

Taxon classificationPlantaeSolanalesSolanaceae

Sw., Syst. Veg., ed. 15 bis [Roemer & Schultes] 4: 647. 1819

27DC880C-03E4-5F4F-B27D-3A21A75403C4

Fig. 2

Solanum kollastrum Gouvêa & Giacomin, PhytoKeys 111: 105. 2018. Type. Brazil. Minas Gerais: Ataléia, povoado de Canaã do Brasil, estrada não pavimentada que liga o município de Ouro Verde de Minas ao povoado de Canaã do Brasil, 18°00'19"S, 41°12'17"W, 313 m alt., Jun 2018, *Y.F Gouvêa 280* (holotype: BHCB [BHCB190863]; isotype: RB [RB1411895, acc. # 787650]).

##### Type.

Brazil. “Ex Brasilia” [probably collected in Mucuri River drainage in the State of Bahia, see discussion], no date, *G.W. Freyreiss s.n.* (lectotype, designated here: S [acc. # S-R-5812]).

##### Description.

Shrubs up to 3.5 m, erect, moderately branched. Stems terete, densely glandular-pubescent and prickly, the trichomes porrect-stellate or somewhat multangulate, sessile to long-stalked, the stalks to 1 mm long, the rays 5–20, 2–3-celled, unequal in length, distally glandular, the mid-point 2–3-celled, equal to or twice the length of the longest ray, distally glandular, the prickles to 1.7 cm long, 2–3 mm in diameter at the base, straight, acicular or only slightly flattened, yellowish-red, basally somewhat pubescent with stellate trichomes like those of the stems and some small, stalked, uniseriate glandular trichomes; new growth densely glandular pubescent with long-stalked stellate or multangulate trichomes and acicular prickles like those of the stems; bark of older stems greyish-dark brown. Sympodial units difoliate to plurifoliate, the leaves not geminate. Leaves simple, lobed; blades (10)20–42 cm long, (8)20–38 cm wide, ca. 1–1.2 times as long as wide, broadly elliptic to broadly ovate, membranous, discolorous, prickly on both surfaces along the veins, the prickles to 1 cm long, straight; adaxial surface densely glandular pubescent, the lamina always visible, the larger trichomes porrect-stellate or multangulate, short- to long-stalked, the stalks to 1 mm long, the rays 4–11, unequal in length, eglandular and 1-celled or 2–3-celled and gland-tipped, the mid-points 2–3-celled, usually longer than the rays, these mixed with smaller sessile to short-stalked porrect-stellate eglandular trichomes, the stalks if present to 0.1 mm long, the rays 2–5, 1-celled, ca. 0.5 mm long; abaxial surface densely glandular-pubescent, the lamina barely visible, the trichomes porrect-stellate to multangulate like those of the adaxial surface, but denser and much more delicate; principal veins 5–7 pairs; sparsely to moderately armed on both surfaces, the prickles 1–1.7 cm long, 1.3–1.8 mm in diameter at the base, straight, somewhat laterally compressed, usually larger abaxially; base cordate, not decurrent, the two major basal lobes 2.5–7 cm long at the longest point, obtuse to rounded, often overlapping each other across the petiole; margins lobed, the lateral lobes 1.5–4.8 cm long, 4–9 cm wide at base, obtuse or rounded or less often acute at the apex, both basal and lateral lobes sometimes with small secondary lobes; apex obtuse or rounded or less often acute; petioles 4.5–19.5 cm long, densely stellate-pubescent with trichomes like those of the stems, usually densely prickly. Inflorescences subopposite the leaves or internodal, 4.5–12 cm long, usually unbranched, rarely forked or trifurcate, with 11–35 flowers, up to 3 open at a time; axes densely glandular-pubescent and prickly, the trichomes porrect-stellate to multangulate, hyaline to yellowish-brown like those of the stems, the prickles ca. 1 mm long, straight, like those of the stems and leaves ; peduncle 2.6–6 cm long; pedicel scars generally unequally spaced, closely packed to spaced 2.3 cm apart; pedicels 4.8–18 mm long, densely pubescent and prickly with trichomes and prickles like those of the stem, but these often purple-tinged, articulated at base. Buds ellipsoid to globose-ellipsoid, the corolla ca. halfway exserted from the calyx tube before anthesis, but enclosed in the calyx lobes. Flowers 5-merous, heterostylous, with basal long-styled co-sexual flowers and functionally staminate short-styled flowers that vary in proportion between inflorescences, the plants andromonoecious. Calyx with the tube 4.5–8.2 mm long, 9.4–15.2 mm in diameter, broadly cup-shaped to somewhat urceolate, inflated, purple-tinged (mainly along the margins and apex of the calyx lobes) to green, armed, densely pubescent with trichomes like those of the stem, but these sometimes purple and with some eglandular rays, the lobes 7.5–15.6 mm long, 6–9 mm wide, triangular, the margins plane to strongly undulate and revolute, the apices acute to caudate. Corolla 2.3–3.9 cm in diameter, purple to lilac or bluish-lilac, white in some stages of development, shallowly stellate to stellate, lobed 2/5 to 1/2 the way to the base, interpetalar tissue absent, the lobes 10.9–15 mm long, 8.8–13.4 mm wide, deltate to triangular, spreading at anthesis, abaxially glandular stellate-pubescent with trichomes like those of the leaves, adaxially almost glabrous with only a few stellate trichomes sparsely distributed along the veins and near the tips, the apex acute, slightly apiculate. Stamens equal; filament tube 1–2.1 mm long; free portion of the filaments 1.3–2.9 mm long, glabrous; anthers 7.5–10 mm long, 2.8–4.3 mm wide, broadly lanceolate and tapering, connivent, glabrous, yellow, abaxially swollen in the lower half (gibbous) and somewhat papillate, poricidal at the tips, the pores directed distally, slightly extrorse, not elongating to slits with age. Ovary conical to somewhat cupuliform, densely stellate-pubescent and glandular at the apex, becoming glabrous with age, the trichomes porrect-stellate, sessile, 2–7-rayed, with a 2–4-celled, eglandular or glandular mid-point longer than the 1-celled rays; style 13.7–15.9 mm long in long-styled flowers, 1.2–3.7 mm long in short-styled flowers, straight, glabrous; stigma large-capitate to clavate, up to 1.4 mm long in long-styled flowers, the surface papillose, green when fresh. Fruit a globose to somewhat compressed globose berry, 1–1.1 cm long, 1.2–2.3 cm wide, pale green to white, glabrous, but with scattered stellate trichomes at the apex, the pericarp somewhat shiny when dry, the berry almost completely enclosed in the saccate fruiting calyx; fruiting pedicels 1.4–2.2 cm long, 1.5–2 mm in diameter at the base, woody and somewhat deflexed from the weight of the fruit, armed with sparse straight prickles like those of the flowering pedicels; fruiting calyx strongly accrescent and inflated, completely enclosing the berry, the tube 1.6–2.1 cm long, 1.9–3.4 cm in diameter at the widest point, the base somewhat plicate and invaginate, the lobes 1.1–2.2 cm long, 1.3–1.9 cm wide, usually somewhat overlapping, densely glandular-pubescent with porrect-stellate to multangulate trichomes. Seeds ca. 230 per berry, ca. 2 mm long, 2.4 mm wide, flattened-reniform, dark brown, the testal cells sinuate in outline; stone cells absent. Chromosome number: not known.

##### Distribution

**Figure 3.**
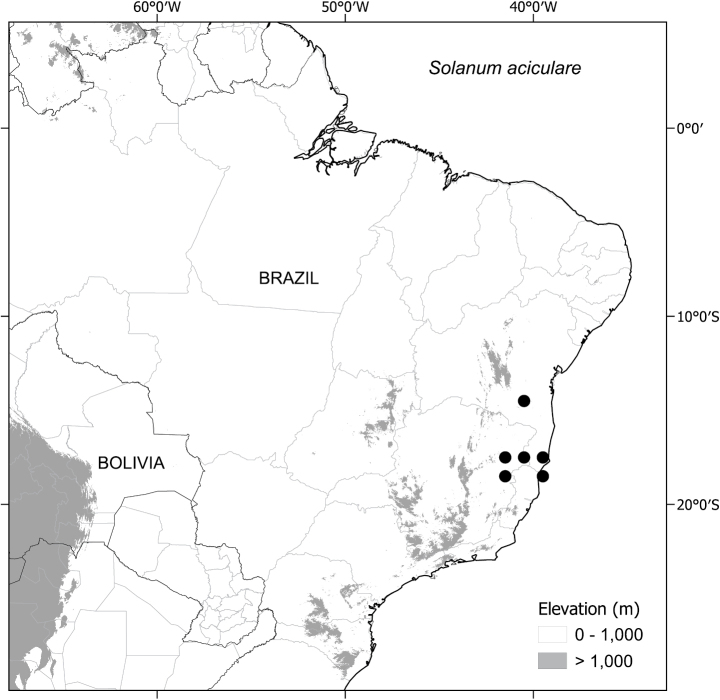
Distribution of *S.aciculare*.

(Fig. 3). *Solanumaciculare* is endemic to eastern Brazil. Records are mostly concentrated along the Mucuri River watershed, ranging from the Municipality of Teófilo Otoni, in north-eastern Minas Gerais State, to Mucuri on the southern coast of Bahia. A single collection (*J.G. Jardim et al. 3151*; CEPEC, NY) is known from further north, in Mun. Caatiba of the south-central region of Bahia State.

##### Ecology.

*Solanumaciculare* inhabits edges of small forest fragments, especially those at the base or on granitic outcrops (inselbergs) or in disturbed sites near these rock outcrops, such as borders of unpaved roads and pastures. It is also found in herbaceous to arboreal vegetation growing along the Brazilian sandy coastal lowlands (restinga *sensu*Araújo (1992)), where plants grow in open disturbed areas dominated by grasses and at the edge of forest fragments (Fig. 2A). Habitats vary from environments subject to periods of drought (e.g. the edge of small seasonal semi-deciduous forest fragments or vegetation islands on inselbergs) to constantly wetter environments, at the edge of coastal evergreen forests, where the climate is under a strong oceanic influence. Plants have been collected from sea level to about 900 m elevation.

##### Common names and uses.

None recorded.

##### Preliminary conservation assessment

(IUCN 2020). EOO (38,277 km^2^, NT); AOO (56 km^2^, EN). Despite the relatively large range of *S.aciculare*, all collections are from only three broad localities and all are outside protected areas; vulnerability of the habitats in which the species occurs and the small number of localities suggest *S.aciculare* should be considered Endangered using the criteria B2 a, b (ii, iii, iv).

##### Discussion.

The name *Solanumaciculare* has not been used previously in treatments of Brazilian *Solanum* (Sendtner 1846) and, until recently, had been considered an ‘unassigned’ name (Flora e Funga do Brazil 2024). Examination of the type specimen and analysis of the collecting trajectory of Georg Freyreiss (see below or in Taxonomy) make clear that this plant is identical to that described as *S.kollastrum* (Gouvêa et al. 2018). Sendtner (1846) had not seen the specimen on which *S.aciculare* was based and his description was of another plant (*Sellow s.n*.) that he admitted did not completely correspond to Swartz’s description (“Solano subscandenti non dissimile in unico specimine suppetente a diagnosi Swartziana paullisper recedens. Proferamus descriptionem nostro specimini accomadatam, momenti, quo discrepat Swarztius haud immemores” - Not dissimilar to Solano subscandens in the only specimen available, departing for a moment from the Swartzian diagnosis. Let us present the description attached to our specimen, the importance of which Swartzius disagrees, not unmindful of it: transl. SK). The specimen described by Sendtner as *S.aciculare* is *S.cordifolium* Dunal, a member of the Erythrotrichum clade.

Amongst the species in this group, only *S.aciculare* and *S.sublentum* have cordate leaf bases coupled with glandular trichomes throughout the stems and leaves. Decurrent leaf bases of *S.aciculare* are only seen in the first leaves of the seedlings, with the subsequent leaves gradually changing shape to become cordate and non-decurrent. In contrast, the leaf bases in *S.hexandrum* and *S.stagnale* remain decurrent throughout plant development, varying in shape from attenuate to truncate.

*Solanumaciculare* closely resembles *S.sublentum*, from which it can be readily distinguished by the robust long-stalked (up to 1 mm) stellate-glandular trichomes with all rays having a glandular distal cell (some rays may lose the glandular cell through breakage or by the disruption of the gland wall) on young stems, petioles and inflorescence axis; trichomes in *S.sublentum* are usually simple. *Solanumaciculare* and *S.sublentum* have very similar floral morphologies, sharing well-developed calyces that are strongly accrescent in fruits, showy purple to lilac corollas and robust anthers. Fruits of *S.aciculare* are completely enclosed in the accrescent calyx, whereas those of *S.sublentum* are exposed. Their leaves also resemble each other: both are lobed (with secondary lobes or not), elliptic to ovate (or broadly ovate in *S.aciculare*) and have cordate bases (varying from truncate to cordate or sagittate in *S.sublentum*). In the field, *S.aciculare* has notably larger leaves than those of *S.sublentum*; however, usually only the apices of the branches are collected, with the fully developed leaves not represented in herbarium material, so this character is often not apparent from herbarium specimens. Although *S.aciculare* and *S.sublentum* occur in similar environmental conditions (associated with outcrops or at edges of lowland forests), they have not been observed in sympatry.

Both *S.aciculare* and *S.phrixothrix* have densely bristly stems and elongate slender inflorescences. They can be distinguished by the glandular long-stalked stellate pubescence of *S.aciculare* (versus eglandular bristles and lack of long-stalked stellate trichomes in *S.phrixothrix*) and flower shape and colour (purple and stellate in *S.aciculare* versus white and rotate in *S.phrixothrix*).

The German collector Georg Freyreiss (also sometimes spelled Freyreis), who was principally an ornithologist and Friedrich Sellow, a botanist, travelled and collected in Brazil in the first decade of the 19^th^ century. Sellow’s botanical collections comprise many hundreds of specimens and were the basis for many new species (Urban 1893; Moraes 2023). In 1815, Freyreiss and Sellow were planning a trip to the north of Rio de Janeiro. They joined forces with Prince Maximilian of Wied, a member of the German aristocracy who was interested in natural history and had been inspired by Baron Alexander von Humboldt to travel to South America, particularly to Brazil (Moraes 2009, 2011). Wied was keen to travel via the, at the time, relatively unexplored coast and, with Freyreiss (Sellow having stayed in what is now the State of Espirito Santo), reached the Rio Mucuri near the border of Espirito Santo and Bahia States, then continued to Salvador in today’s State of Bahia. The type specimen of *S.aciculare* was probably collected by Freyreiss in the Mucuri River drainage during this voyage.

#### ﻿2. Solanum hexandrum

Taxon classificationPlantaeSolanalesSolanaceae

Vell., Fl. Flumin. 88. 1829.

D5616772-7101-5E55-BB3E-501BB86B2620

Figs 4 , 5

Solanum hexandrum Vell. var. minax Sendtn., Fl. Bras. (Martius) 10: 71. 1846. Type. Brazil. São Paulo: “primaevis sulvis supra Serra do Mar, Prov. Sebastianopolit.”, Dec, *C.F.P. van Martius s.n.* (lectotype, designated here: M [M-0171650]).
Solanum maroniense Poit. var. hexandrum (Vell.) Dunal, Prodr. [A. P. de Candolle] 13(1): 319. 1852. Type. Based on Solanumhexandrum Vell.
Solanum echidniforme Dunal, Prodr. [A. P. de Candolle] 13(1): 324. 1852, as "echidnaeforme". Type. Brazil. Sin. loc., *J. Lhotsky s.n.* (holotype: G-DC [G00131228]).
Solanum polytrichum Moric. var. enoplocalyx Dunal, Prodr. [A. P. de Candolle] 13(1): 324. 1852. Type. Brazil. Rio de Janeiro: Serra dos Orgãos [“circa de Rio de Janeiro” – protologue], *C. Gaudichaud 500* (lectotype, second step designated here; first step designated by Nee 1996, pg. 32 [as “holotype”]: P [P00368655]; isolectotype: P [P00368656]).
Solanum maroniense Poit. forma hexandrum (Vell.) Voss, Vilm. Ill. Blumengartn., ed. 3, 1: 719. 1894. Type: Based on Solanumhexandrum Vell.

##### Type.

Brazil. [Rio de Janeiro]: “habitat silvis nondum cultis”; (lectotype, designated by Knapp et al. (2015), pg. 831: [illustration] Original parchment plate of Flora Fluminensis in the Manuscript Section of the Biblioteca Nacional, Rio de Janeiro [cat. no.: mss1198651_125] and later published in Vellozo, Fl. Flumin. 2: tab. 122. 1831).

##### Description.

Shrubs (0.5-)1–2.5 m tall, erect or occasionally somewhat spreading, strongly armed. Stems terete, glabrous to densely pubescent and/or bristly, sparsely to densely prickly, the stellate trichomes long-stalked, eglandular, porrect-stellate or less often appearing simple due to the complete lack of rays, the stalks 1–5 mm long, multiseriate, the rays 4–7, ca. 1 mm long, the mid-point ca. 0.5 mm long, always shorter than the lateral rays, with age the trichomes becoming thicker and the stems then densely bristly, the bristles often tipped with stalks and rays, underlying pubescence of minute papillate trichomes dense, more apparent on more glabrous individuals, the prickles 0.3–2 cm long, yellowish-golden, broad-based, the base 1.5–2 mm in diameter; new growth glabrous to densely stellate-pubescent and bristly, the multiseriate stalks of stellate trichomes usually shorter than the rays, but lengthening with leaf expansion; bark of older stems dark brownish-black in herbarium specimens, dark brown in live plants. Sympodial units unifoliate or difoliate, the leaves not geminate. Leaves simple or shallowly lobed (in some specimens, for example, *de Paula 641*, *Giacomin 1827* deeply lobed) and repand, the blades 12–35(-40) cm long, 8–26(-30) cm wide, ca. 1.3–1.5 times as long as wide, broadly elliptic to narrowly obovate, usually widest in the basal half, membranous, concolorous, usually prickly on both surfaces with scattered straight prickles to 0.4–1.5 cm long, the prickles occasionally absent; adaxial surface glabrous to sparsely to moderately and evenly pubescent with long-stalked porrect-stellate trichomes, the stalks 1–1.5 mm long, multiseriate and arising from an expanded base, the rays 4–7, 1–2 mm long, the mid-point 0–1 mm long, always shorter than the rays, in some individuals, the rays often lost and the trichomes then appearing to be composed of a multiseriate base 1–1.5 mm long with a single celled tip often bent at 90° to the leaf surface; abaxial surfaces glabrous to evenly and densely pubescent with similar porrect-stellate long-stalked trichomes, but the stalks thinner and shorter than those on the adaxial surfaces and the rays occasionally more numerous, the trichomes denser along the veins, the surface densely dotted with crystal sand (inclusions of calcium oxalate, this not visible on the upper surfaces); principal veins 4–6 pairs, prickly or not, the prickles, if present, 0.4–1.5 cm long, on both surfaces; base attenuate on the petiole with a wing of ca. 2 mm wide along half the petiole length, sometimes to base of petiole; margins entire to 6-lobed, the lobes usually shallow, the sinuses less than 1/4 of the distance to the mid-rib; apex acute to attenuate; petiole (0.1-)2–10 cm long, glabrous to densely stellate pubescent and bristly, usually sparsely prickly. Inflorescences opposite the leaves or internodal, (1.5-)2.5–6(-8) cm long, unbranched (rarely furcate), with 3–10 flowers; axes glabrous to densely stellate-pubescent and prickly like the rest of the plant, the bristles and trichomes grading into each other and not distinct in morphology; peduncle (0.5-)2–7 cm long; pedicels 1–2 cm long, 1–1.5 mm in diameter at the base, 1.5–2 mm at the apex (excluding trichomes), erect to spreading, glabrous to densely stellate-pubescent and bristly, if prickly, the prickles ca. 1 mm long and thinner than those of stems and leaves, articulated at the base; pedicel scars more or less evenly spaced 5–7 mm apart on mature inflorescences, more tightly packed distally. Buds globose to broadly elliptic, the corolla completely included in the saccate calyx tube until just before anthesis, the younger buds less bristly and prickly than older ones. Flowers 5–6-merous, mostly co-sexual, but a few distal flowers are sometimes short-styled and probably functionally staminate. Calyx with the tube 4–7 mm long, 7–10 mm in diameter, cup-shaped and often completely closed in bud sometimes until just before anthesis, green or purple-tinged, the lobes 5–10 mm long, irregularly tearing at anthesis, but generally broadly triangular to deltate, acute to acuminate apically, glabrous to bristly and prickly with long-stalked bristles/trichomes, these with or lacking rays, the multiseriate stalk often purple-tinged. Corolla 3–6 cm in diameter, purple (rarely white), stellate, lobed halfway to the base, interpetalar tissue thin, glabrous, the lobes 15–21 mm long, 8–15 mm wide, deltate, spreading at anthesis, abaxially sparsely to densely pubescent with long-stalked porrect stellate trichomes, the stalks to 1 mm long, these denser at the tips and along the petal mid-vein, pubescence of corollas often purple-tinged, adaxially glabrous or with a few weak stellate trichomes on the mid-vein, the mid-veins often white adaxially. Stamens equal; filament tube minute to 0.5 mm long, glabrous; free portion of the filaments 1–1.5 mm long, glabrous; anthers (7-)9–11 cm long, 2–3 mm wide, broadly lanceolate and tapering, connivent, glabrous, yellow, abaxially swollen in the lower half (gibbous) and somewhat papillate, poricidal at the tips, the pores directed distally, not elongating to slits with age. Ovary conical, sparsely to densely pubescent with sessile stellate trichomes with rays 2–3 mm long, these soon deciduous; style 10–15 mm long in long-styled flowers (in rare short-styled flowers, the style 3.5–7 mm long), straight, glabrous; stigma clavate or broadly capitate, the surface minutely papillose. Fruit a globose to flattened globose berry, 2–2.5(-3.5) cm in diameter, green or pale whitish-green, glabrous, the pericarp somewhat shiny when dry, the berry almost completely enclosed in the accrescent saccate calyx; fruiting pedicels 1.8–2.3 cm long, 1.7–2.5 mm in diameter at the base, woody and spreading to somewhat deflexed from the weight of the fruit; fruiting calyx strongly accrescent, inflated or not, almost completely enclosing the berry, the tube 1.5–2 cm long, the lobes 1.5–2 cm long, irregular, usually overlapping, glabrous to sparsely to densely bristly and prickly with multiseriate bristles occasionally topped with porrect rays. Seeds ca. 100 per berry, 2–3.5 mm long, 1.5–2 mm wide, flattened reniform to somewhat ovoid, unwinged, reddish-brown or dark brown when dry, the surface minutely pitted, the testal cells pentagonal in outline, equal in size over the entire seed surface; stone cells absent. Chromosome number not known.

**Figure 4.**
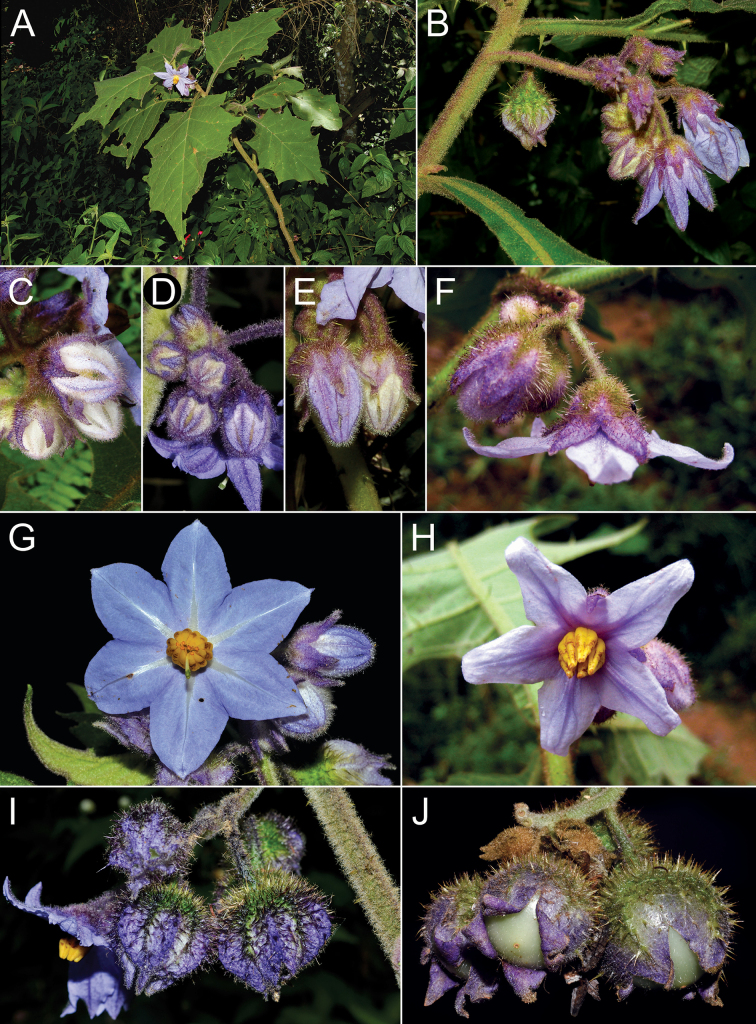
*Solanumhexandrum* (pubescent individuals) **A** habit **B** inflorescence **C–E** buds showing differences in colour and shape **F** open flower **G** long-styled flower with 6 lobes **H** short-styled flower with 5 lobes **I** developing fruits enclosed in purple-tinged calyx **J** mature berries with tightly appressed accrescent calyx (**A**, **B***Stehmann et al. 4513*; **C***Giacomin et al. 875*; **D**, **G**, **I***Agra et al. 7359*; **E**, **F**, **H***Giacomin et al. 1827*; **J***Gouvêa & Salino 514*). Photos: **A, B** João R. Stehmann **D**, **G**, **I** Leandro L. Giacomin **E**, **F**, **H** Lynn Bohs **J** Yuri F. Gouvêa.

**Figure 5.**
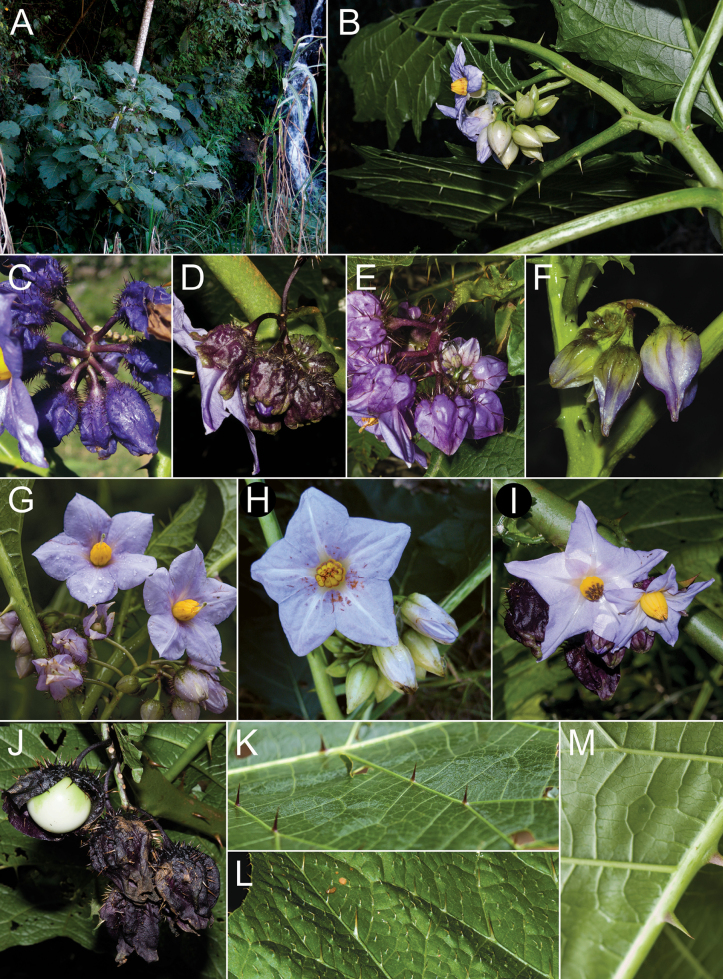
*Solanumhexandrum* (glabrous individuals) **A** habit at forest edge **B** inflorescence **C–F** buds showing variation in fusion of calyx during development **G** open long-styled flowers **H** open short-styled flower **I** open short-styled flowers showing corolla expansion (enlargement) during flowering **J** mature berry with calyx removed **K** upper leaf surface with prickles and no stellate trichomes **L** upper leaf surface with prickles and rayless stellate trichomes with bent mid-points **M** lower leaf surface with broad-based prickles along the veins (**A**, **B**, **H***Giacomin et al. 1833*; **C***Giacomin et al*. *1844*; **D, I, J***Gouvêa & Falcão 137*; **E**, **L***Gouvêa & Falcão 135*; **F***Gouvêa & Stehmann 158*; **G, K, M***Gouvêa & Stehmann 159*). Photos: **A, C** Lynn Bohs **B**, **H** Leandro L. Giacomin **D**, **E**, **I**, **J**, **L** Yuri F. Gouvêa **F**, **G**, **K**, **M** João R. Stehmann.

##### Distribution

**Figure 6.**
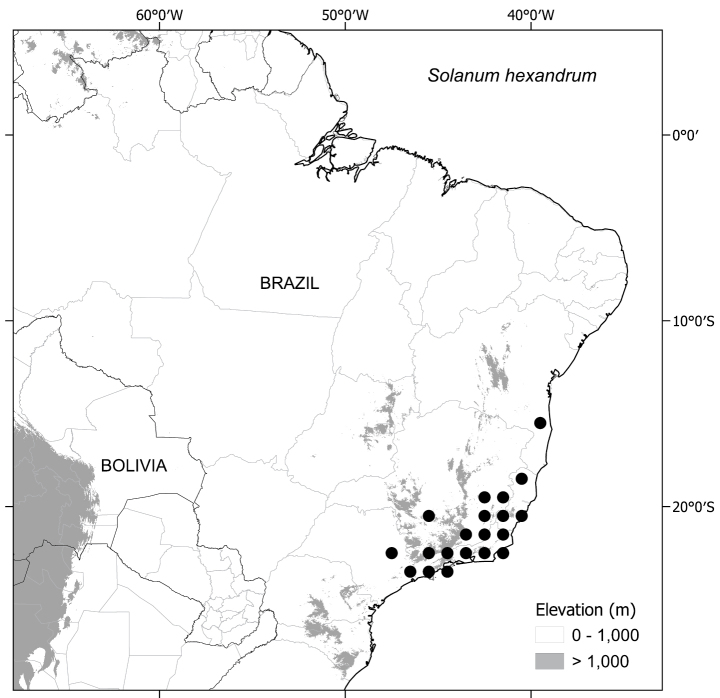
Distribution of *S.hexandrum*.

(Fig. 6). *Solanumhexandrum* is endemic to the south-eastern region of Brazil and is known from the States of São Paulo, Rio de Janeiro, Minas Gerais, Espirito Santo and Bahia.

##### Ecology and habitat.

*Solanumhexandrum* grows in the wet forests of the Mata Atlântica, often in openings and along roads and streams; it occurs from almost sea level to 1,600 m elevation.

##### Common names and uses.

Brazil. Minas Gerais: juá-bravo (a widely used vernacular name for any spiny solanum in Brazil). No uses have been recorded.

##### Preliminary conservation status

(IUCN 2020). EOO (327,280 km^2^, LC); AOO (460 km^2^, EN). *Solanumhexandrum* is the most collected of any of these species and all forms of the variation are known from many localities, several of which are within protected areas (e.g. Parque Nacional da Serra dos Orgãos, Parque Nacional do Caparaó, Parque Estadual da Pedra Selada in Rio de Janeiro State; Parque Estadual da Serra do Brigadeiro in Minas Gerais State; RPPN Cafundó, Parque Estadual Mata das Flores in Espírito Santo State; RPPN Serra do Teimoso and RPPN Serra Bonita in Bahia State; Estação Ecológica de Bananal in São Paulo State). Nevertheless, given its high degree of variability that needs further study, we suggest it be assigned a preliminary status of Near Threatened based on criteria (B 2 a, b (ii,iii,iv).

##### Discussion.

*Solanumhexandrum* is the most variable species in the clade in terms of calyx and corolla shape and degree of pubescence, but is otherwise remarkably uniform. More glabrous individuals from the more northerly part of the species range have been called *S.echidniforme* Dunal, although the type of that species (G00131228) is a sparsely bristly plant and fits within the overall circumscription as we treat this species here. The more glabrous individuals of *S.hexandrum* (mostly from the State of Espirito Santo) are strikingly different looking morphologically from more pubescent individuals (see Fig. 5), but there is a continuous gradation from glabrous to densely pubescent when specimens from across the range are examined. In several localities, individuals of both types are found and it is not clear if the differences are due to environmental or genetic factors.

The pubescence variability in *S.hexandrum* is extreme, with glabrous and pubescent individuals at first glance appearing to be completely different morphologically. Some other variation seems to be correlated with lack of rayed stellate trichomes; glabrous plants often have the calyx lobes fused until just before anthesis (see Fig. 5B, D), but this is not completely consistent (see Fig. 5C). The calyx in these glabrous plants is also often more saccate than in populations from elsewhere in the range (Fig. 5J versus Fig. 4J), but this also varies and is difficult to assess on herbarium sheets when all reproductive stages are not present. The single specimen of *S.hexandrum* which we have seen with a branched inflorescence (*Brotto et al. 3265*, MBM) comes from amongst these glabrous plants.

The unusual stellate trichomes with the single-celled mid-point bent at an approximately 90° angle to the multiseriate base (see Fig. 5L) are found on the leaves in many populations of *S.hexandrum*, even if very sparsely. Leaf shape also varies across the range, with some populations from Rio de Janeiro having very narrow, more deeply lobed leaves (e.g. *Giacomin 1827*). The range of variation in trichome morphology in *S.hexandrum* warrants further study at a population and genetic level to determine if these variants represent distinct entities. Loss of prickles (derived from stellate trichomes) has been shown to be common across spiny solanums (Satterlee et al. 2024) and it may be that the extremes seen in *S.hexandrum* could be genetically quite simple.

*Solanumhexandrum* was described and illustrated in the late 18^th^ century by Brother José Mariano Conceição da Vellozo (1742–1811) for his "*Flora Fluminensis*". Vellozo was a parish priest in Rio De Janeiro and completed his work in 1790, but, unfortunately, this work was not published until long after he had died in 1811 (Carauta 1969, 1973). The somewhat telegraphic descriptions of *Solanum* (Vellozo 1829) referred to original illustrations now held in the library of the Biblioteca Nacional, Rio de Janeiro; printed illustrations, based on these originals, were published several years later (Vellozo 1831). Typification of names of Solanaceae in *Flora Fluminensis* was treated by Knapp et al. (2015); the original illustration that is the lectotype for *S.hexandrum* is unambiguous and clearly shows the 6-parted corolla that inspired the specific epithet.

Many herbarium specimens of *S.hexandrum* are annotated as *S.maroniense* Poit., a species described only a year after (Poiteau 1830). The description of *S.maroniense* is of a cultivated plant and is quite telegraphic, but is clearly stated to come from “fleuve Maroni” (the River Maroni) in French Guiana. Although, from the description alone, *S.maroniense* could correspond to *S.hexandrum*, the locality suggests it represents a plant of *S.crinitum* Lam. *Solanumcrinitum* is common in the Guianas and a neotype will be selected as part of upcoming monographic work on the Crinitum clade.

Sendtner (1846) described a variety (“β minax”) citing a manuscript name of Martius, citing several specimens from various collectors, as well as plants from the garden in Munich. A sheet at Munich (M-0171650) exactly matches the very precise locality information and date given in the protologue for a specimen collected by C.F.P. von Martius and we select this as the lectotype; it is the most unambiguous of the syntypes and appears to have been annotated by Sendtner. Another sheet at Munich (M-0171847) has a description and the annotation “Solanumminax” in Martius’ hand is probably also a syntype; this sheet was collected in “Oct” while the lectotype was collected in “Dec” – the protologue states “Octobri ad Decembrem florens”. Other collections cited have no or less unambiguous localities (see Suppl. materials 1, 2). Sendtner (1846) suggested that *S.latifolium* Poir. (= *S.rigidum* Lam.) might be a synonym of *S.hexandrum*; this species is endemic to the Cape Verde Islands and related to the brinjal eggplant (Knapp and Vorontsova 2013).

Dunal (1852: 324) cited *Gaudichaud 501* from Rio de Janeiro in “h. Mus. Paris” in his circumscription of S.polytrichumMoric.var.longifolium along with *Blanchet 602* from Bahia in “h. DC”. Solanumpolytrichumvar.longifolium is illegitimate and superfluous as he (Dunal 1852: 324) cited an earlier name at the varietal level, S.polytrichumvar.grandifolium Sendtn. (Sendtner 1846), in synonymy. The three specimens of *Gaudichaud 501* in P (P00368657, P00368658, P00368659) are of a plant of *S.hexandrum* and, although P00368657 has been annotated as an “Isosyntype of Solanumpolytrichum Moric. var. longifolium Dunal”, this collection has no status as a type. Both collections (*Gaudichaud 500*, 501) cited by Dunal (1852) may actually have been collected by Frederich Sellow (see Moraes (2023)).

*Gaudichaud 500* in “h. Mus. Paris” was the only element cited in the protologue of S.polytrichumvar.enoplocalyx (Dunal 1852). Nee (1996) cited as “holotype” *Gaudichaud 500* in P and cited two photographs taken by C.V. Morton (Morton neg. 8300, 8301, both held in US). Two sheets of this collection are in P, one bears the original locality label (P00368655), while the other (P00368656) has an undated label in what appears to be Dunal’s hand. Both are scrappy specimens with tiny apical leaves, but P00368655 has two inflorescences, one in flower and one in early fruit. As the more complete specimen, we select the sheet P00368655 of *Gaudichaud 500* as the second step lectotype for S.polytrichumvar.enoplocalyx. This sheet is labelled as an isotype. Both of these sheets are very similar morphologically to those labelled *Gaudichaud 501* and may have been collected together.

#### ﻿3. Solanum hydroides

Taxon classificationPlantaeSolanalesSolanaceae

Gouvêa & Giacomin, PhytoKeys 139:66. 2020.

0DE590B3-446C-5095-92EF-6621DEC44D21

Fig. 7

##### Type.

Brazil. Minas Gerais: Mun. Teófilo Otoni, afloramento rochoso lado esquerdo da MG 418, cerca de 30 km norte de Teófilo Otoni, 560 m alt., 17°51'22"S, 41°15'39"W, 27 Jan 2014, *L.F.A. de Paula*, *L. Azevedo*, *R. Fernandes & J.R. Stehmann 669* (holotype: BHCB [BHCB053358] ; isotype: RB [RB01472905]).

##### Description.

Shrubs 1–1.5 m tall, erect, armed. Stems terete, directed upwards and spreading, moderately to densely pubescent and sparsely to moderately prickly, the trichomes eglandular, porrect-stellate, variably short- to long-stalked, the stalks 0.5–1.5 mm long, the rays 4–8, 0.5–1 mm long, the mid-points 1– to 2–celled, always shorter than the rays, the prickles 4–6 mm long, 2–6 mm wide at the base, broad-based and recurved; new growth densely stellate pubescent and sparsely prickly, the trichomes pale yellow to dark brownish-red; bark of older stems glabrescent, drying greenish-brown to dark brown. Sympodial units plurifoliate, the leaves not geminate. Leaves simple, nearly entire to shallowly lobed; blades 2.8–12.1(21.8) cm long, 2.2–7.5(10.1) cm wide, ca. 1.2 to 2 times as long as wide, elliptic to ovate, membranous, slightly discolorous, both surfaces prickly along the mid-rib and veins; adaxial surface densely to moderately stellate-pubescent and prickly, brown to dark green when dry, the trichomes like those of the stem, but with (1–)4–6 rays, the prickles along the mid-rib and major veins to 5.5 mm long and 1 mm wide at the base, straight and laterally compressed; abaxial surface more densely stellate-pubescent than the adaxial surface, whitish-green when dry, the trichomes like those of the adaxial surface, the prickles like those of the adaxial surface, but to 6.5 mm long and 2 mm wide at the base; base attenuate to truncate or rounded, less often with 1 or 2 basiscopic lobes, decurrent on to the petiole, sometimes asymmetrical; margins shallowly lobed, the lobes (0)3–5 on each side, 1–12(14.8) mm long, 3.2–11(23) mm wide at base with usually acute, sometimes rounded or obtuse apices, the sinuses 3.2–8.5 mm deep; apex acute to acuminate; principal veins 4–6 pairs, more prominent beneath, prickly on both surfaces, the prickles 5–6 mm long, straight; petioles 0.6–3.3 cm long, densely to moderately pubescent with porrect-stellate trichomes like those of the leaves, usually armed with 1–5 prickles. Inflorescence internodal, to 6 cm long, unbranched, with 4–10 flowers, up to 2 flowers open at a time; axes glabrescent to densely pubescent, usually unarmed, the stellate trichomes like those of the stem, but these sometimes with the mid-point as long as the rays; peduncles 0.4–2.3 (-3.8) cm long; pedicels 3–17 mm long, 0.5–0.8 mm in diameter at the base, to 1.5 mm in diameter at the apex, spreading to slightly deflexed, pubescent with trichomes like those of the inflorescence axes, unarmed, articulated at the base; pedicel scars evenly spaced 1–7 mm apart. Buds ovoid to ellipsoid, with the corolla enclosed in the calyx until just before anthesis. Flowers 5-merous, heterostylous with long-styled flowers (co-sexual) at the base of inflorescence, short-styled (functionally staminate) flowers more distally, the plants andromonoecious. Calyx with the tube 2.6–4.3(6) mm long, 6.5–8 mm in diameter, broadly obconical to cupuliform, the lobes 3–7 mm long, 3–5.5 mm wide, triangular to deltate, with acute to acuminate apices, glabrous adaxially, densely pubescent abaxially with bristly purple-tinged, hyaline or reddish-brown porrect to multangulate long-stalked stellate trichomes, the stalks 1.1–3.8 mm long, rays 4–8, to 1.5 mm long, the mid-points 1–2 celled, shorter than or the same length as the rays, armed or unarmed, if present, the prickles 2.8–4 mm long, 0.5–1 mm in diameter at the base, straight, acicular. Corolla 2.4–3 cm in diameter, white, often with a greenish-yellow star at the base, shallowly stellate, lobed ca. halfway to the base, interpetalar tissue nearly absent, the lobes 5.9–8.8 mm long, 9.9–12.2 mm wide, pubescent abaxially on the petal mid-vein and/or apices with sparse delicate short-stalked porrect-stellate trichomes with stalks to 0.9 mm long, the apices acute to apiculate. Stamens equal; filament tube to 1 mm long; free portion of the filaments 0.7–1 mm long, glabrous; anthers 6.5–8 mm long, 2.5–3 mm wide, broadly lanceolate and tapering, connivent or slightly divergent at the tips, glabrous, yellow, abaxially swollen in the lower half (gibbous) and somewhat papillate, poricidal at the tips, the pores directed distally, slightly extrorse, not lengthening to slits with age. Ovary somewhat conical, glabrous; style 8–10 mm long in long-styled flowers, ca. 3 mm long in short-styled flowers, straight, glabrous; stigma clavate to bilobed, the surface papillose and irregular, the style and stigma poorly developed in short-styled flowers. Fruit a globose berry, 0.9–1.8 cm in diameter, green to whitish-green at maturity, drying dark brown, glabrous, the pericarp matte; fruiting pedicels 1–1.5 cm long, 1–2 mm in diameter at the base, usually unarmed, deflexed from the weight of the fruit; fruiting calyx partially accrescent, the tube tightly investing 1/2–3/4 of the fruit at maturity, the lobes 5.8–8 mm long, 7–9.6 mm wide, not overlapping, pubescent with long-stalked porrect-stellate trichomes often with the base of the stalks markedly expanded and bristly, the stalks to 4.8 mm long. Seeds ca. 250 per berry, 2.2–2.6 mm long, 1.6–2 mm wide, pyriform to reniform, not markedly flattened, the surface irregularly pitted, the testal cells pentagonal in outline; stone cells absent. Chromosome number not known.

**Figure 7.**
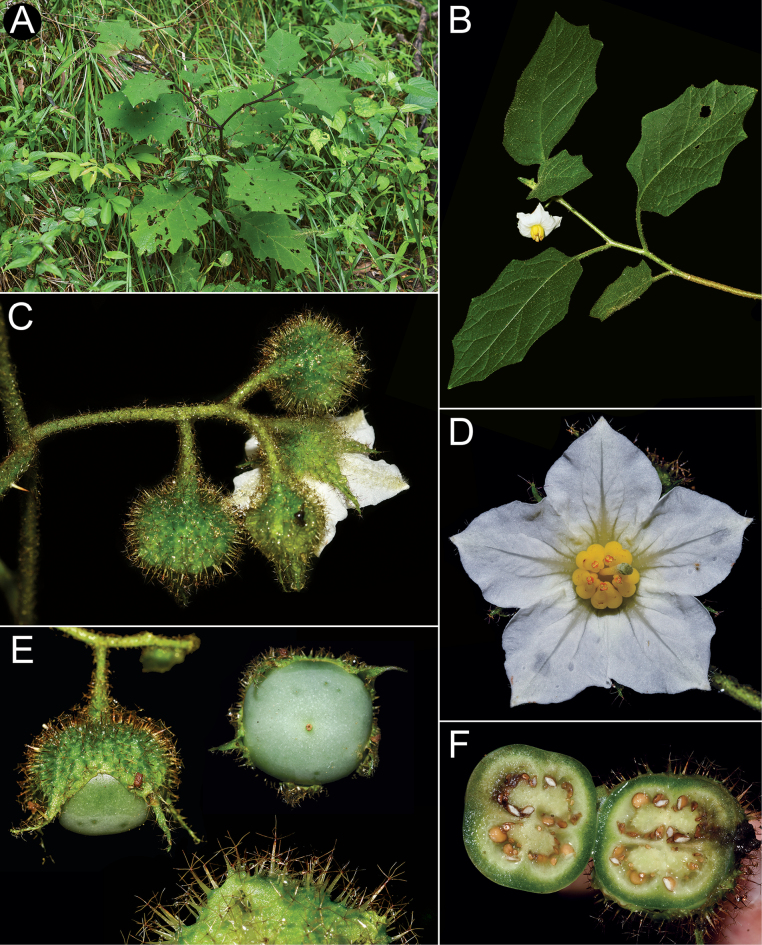
*Solanumhydroides***A** habit **B** flowering branch **C** inflorescence **D** open long-styled flower **E** mature berry with only partially accrescent calyx **F** cross-section of 4-locular mature berry showing fleshy mesocarp and brown seeds (**A**, **C–F***Gouvêa et al. 492*; **B***Gouvêa & Santos 325*). Photos: Yuri F. Gouvêa.

##### Distribution

**Figure 8.**
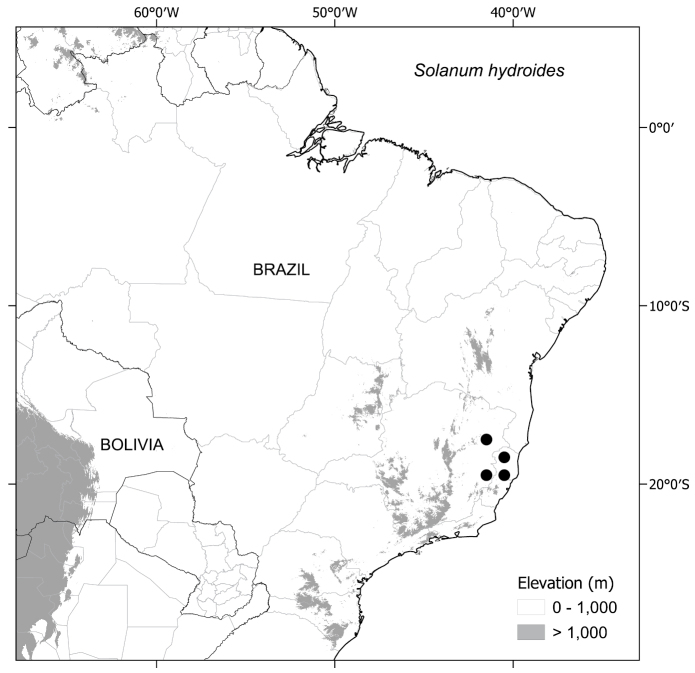
Distribution of *S.hydroides*.

(Fig. 8). *Solanumhydroides* is endemic to the south-eastern region of Brazil, with records in four localities in north-eastern Minas Gerais (Mun. Teófilo Otoni and Conselheiro Pena) and northern (Mun. Nova Venécia) and central (Mun. Santa Teresa) Espírito Santo States.

##### Ecology and habitat.

*Solanumhydroides* grows at the edge of seasonal semi-deciduous tropical rainforests associated with granitic or gneissic rock outcrops (inselbergs) and somewhat disturbed sites at their bases, such as roadsides and clearings; from 300 to 600 m elevation. It also occasionally grows in epilithic vegetation patches lying on the flatter parts of inselbergs.

##### Common names and uses.

None recorded.

##### Preliminary conservation status

(IUCN 2020). EOO (12,549 km^2^, VU); AOO (24 km^2^, EN). *Solanumhydroides* is known from only four disjunct localities in vegetation remnants associated with inselbergs: these rock outcrops harbour the last remnants of forest fragments (Martinelli 2007) in areas where they occur. Only one of these localities is within a protected area (APA Pedra do Elefante, Espiríto Santo State). Although we have seen a few more collections than were used in the original assessment (Gouvêa et al. 2020), we concur with their evaluation of *S.hydroides* as Endangered (B 2 a,b ii,iii,iv).

##### Discussion.

*Solanumhydroides* is a comparatively smaller plant than other species of the group, except *S.sublentum*; its smaller leaves and thinner stems, petioles and inflorescence axes give it a more delicate overall aspect. *Solanumhydroides* can, however, be readily distinguished from *S.sublentum* by its pubescence of stellate eglandular trichomes (Fig. 7A, B) and by the widely obconical to cupuliform shape of the calyx at anthesis. In *S.sublentum*, the indumentum is of both conspicuous simple glandular trichomes and stellate eglandular trichomes (Fig. 13E–G), with the stellate trichomes usually much less numerous than the simple ones and often early deciduous (i.e. present only in new growth). Calyces of *S.sublentum* are somewhat urceolate, inflated and prominently plicate at the top of the calyx tube (Fig. 13J), whereas, in *S.hydroides*, calyces are tightly adherent to the berry at maturity and not notably plicate, especially in live plants.

Although being a markedly less robust plant, *S.hydroides* can be very similar to some specimens of *S.hexandrum*, the most variable species in the clade, with which it shares the indumentum of few-rayed stellate eglandular trichomes on the stems, leaves, inflorescence axis and calyces. *Solanumhydroides* differs from *S.hexandrum* in its white and smaller corollas (13–21.5 mm total length), shorter corolla lobes (5.9–8.8 mm long; Fig. 7D) and accrescent, but not inflated, fruiting calyces that only partially cover the mature fruit (Fig. 7E). *Solanumhexandrum* has corollas in various shades of lilac to purple and are larger (24.3–40 mm long), with longer corolla lobes (12.6–25 mm long; Figs 4G, H, 5G, H, I) and the fruiting calyces are accrescent and inflated, completely enclosing the mature fruit (Figs 4J, 5J). The corollas of *S.hydroides* are thin and membranous and easily tear apart between the lobes during the drying process, which can make the lobes on herbarium specimens seem longer than they really are. Care is needed to ensure correct measurements from herbarium specimens.

Leaf measurements are also useful for distinguishing *S.hydroides* from *S.hexandrum*. The leaves of *S.hydroides* are usually smaller (7.5–13.6 cm long and 5–8.7 cm wide) than those of *S.hexandrum* (17–45 cm long and 10.5–32 cm wide). Nevertheless, leaves of *S.hydroides* are larger in plants growing in shade and in young individuals (see Roe (1966) for other examples in *Solanum*) and we have seen plants with leaves to 22 cm long and 11 cm wide. Specimens of *S.hydroides* growing in shade are less densely pubescent, with less robust (i.e. stalks with fewer series of cells) and slightly shorter trichomes on stems, leaves and calyx. Corollas of these shade plants are usually larger in relation to the other flower parts (e.g. stamens and calyx).

Trichome morphology in *S.hydroides* is not particularly variable within individual plants and amongst plants of the same population; however, there is a significant variation in the number of trichome rays between some populations, as is seen also in *S.hexandrum*. Trichomes of specimens from the southernmost-known population (in Santa Teresa Municipality, Espirito Santo State) are mostly 6- to 8-rayed and usually denser, whereas those of plants from the other populations (Teófilo Otoni and Nova Venécia Municipalities of Minas Gerais State) are mostly 4-rayed. Within individual plants, the variation in trichome morphology is limited to a reduction in the number of rays and is especially evident in plants with four-rayed trichomes. In these plants, the trichomes may lack one to almost all rays, sometimes with only the mid-point or a lateral ray remaining and the trichome appearing to be unbranched, but with a basal multiseriate stalk, as is also seen more dramatically in *S.hexandrum*. This kind of variation has been reported in other *Solanum* groups, such as the Brevantherum clade or members of the Acanthophora clade (Nee 1991; Levin et al. 2005; Stern et al. 2013).

#### ﻿4. Solanum phrixothrix

Taxon classificationPlantaeSolanalesSolanaceae

Gouvêa & S.Knapp sp. nov.

5EF90468-007D-5F1B-8334-5821A4CB995E

urn:lsid:ipni.org:names:77358212-1

Fig. 9

##### Diagnosis.

*Solanumphrixothrix* differs from all other members of the *S.hexandrum* group in its rotate, white corollas. It is similar to *S.aciculare* and *S.sublentum* in its cordate non-decurrent leaf bases, but differs from both in its eglandular pubescence. The eglandular bristle-like trichomes completely lacking lateral rays distinguish it from *S.aciculare* and its densely bristly stems distinguish it from *S.sublentum*. It differs from *S.hexandrum*, *S.hydroides* and *S.stagnale* in its cordate non-decurrent leaf bases and from *S.stagnale*, it is acicular, rather than broad-based and usually curved prickles.

**Figure 9.**
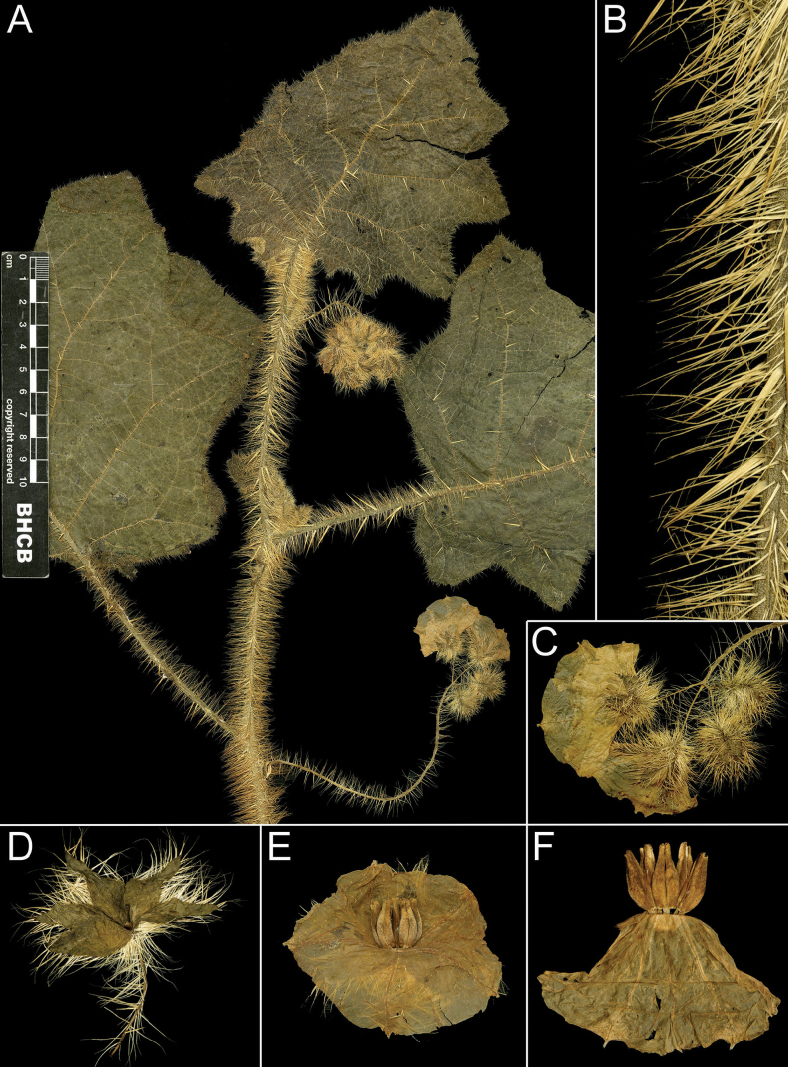
*Solanumphrixothrix***A** flowering branch **B** section of stem with copious bristles **C** inflorescence with two open flowers **D** adaxial surface of calyx **E** open short-styled flower **F** short-styled flower with corolla bent back to expose the tapering anthers (**A–F***Folli 7560*, BHCB). Photos: Yuri F. Gouvêa.

##### Type.

Brazil. Espirito Santo: Mun. Linhares, bairro Bebedouro, mata de tabuleiro, estrada ES-245, sentido a Regência, mata de cabruca (Cacau), UTM: 394627, 7851005 [19°25'57.1"S, 40°00'13.5"W], 12 Jun 2017, *D.A. Folli 7560* (holotype: BHCB [BHCB221244]; isotype: CVRD [acc. # 15743]).

##### Description.

Shrubs to ca. 2 m tall, erect to somewhat spreading, strongly armed and bristly. Stems terete, conspicuously fistulose (at least the younger ones), densely bristly and prickly; the bristle-like trichomes simple, 3.4–19.2 mm long, with a long multiseriate stalk and a shorter uniseriate mid-point at the tip, the stalks 2.5–16 mm long, the mid-points 0.9–3.2 mm long, 1–2-celled, underlying pubescence of sparse, very tiny papillate trichomes, these drying golden yellow; the prickles 0.5–1.2 cm long, the base ca. 1 mm in diameter, straight to retrorse, somewhat laterally compressed, yellowish-golden at base, becoming yellowish-brown towards the tip; the epidermis densely dotted with crystal sand (inclusions of calcium oxalate); new growth densely bristly and prickly, with simple bristle-like trichomes and prickles like those of the stems; bark of older stems not known. Sympodial units plurifoliate, the leaves not geminate. Leaves lobed, the blades 21–30 cm long, 20–24 cm wide, ca. 1–1.25 times as long as wide, broadly ovate to broadly elliptic, usually widest in the basal half, membranous, concolorous, sparsely to moderately prickly on the mid-rib and major veins of both surfaces with prickles like those of the stems, but usually smaller; adaxial surface densely to moderately pubescent to hirsute with simple bristle-like trichomes similar to those of the stems, but smaller, 0.8–6.5 mm long, the stalks 0.2–4.5 mm long, the mid-points 0.7–2 mm long; abaxial surfaces more sparsely pubescent with trichomes like those of the adaxial surface, these restricted to the mid-rib, major and minor veins and usually smaller and thinner-walled; principal veins 5–7 pairs; base cordate to angular-cordate often with a prominent pair of basiscopic lobes, not decurrent on to the petiole, symmetrical; margins 4–6-lobed, leaves of young plants or new growth also with secondary lobing, the sinuses 1/3–1/5 of the distance to the mid-rib; apex acute to obtuse; petiole 4.8–9.5 cm long, densely bristly, moderately to densely prickly with trichomes and prickles like those of the stems. Inflorescences opposite the leaves or internodal, 12–15 cm long, unbranched, with 19–25 flowers; axes densely to moderately bristly and prickly with trichomes and prickles like those of the stems, but the prickles sometimes thinner; peduncle 2.7–3.6 cm long; pedicels 1–2 cm long, 0.6–0.9 mm in diameter at the base, same diameter at the apex (excluding trichomes), erect to directed downwards, moderately to densely bristly with bristle-like trichomes like those of the stems, prickly or not, articulated at the base; pedicel scars more or less evenly spaced 1–5 mm apart on mature inflorescences, more closely packed distally. Buds ellipsoid to narrowly ellipsoid, the calyx lobes soon splitting, exceeding the length of the corolla until just before anthesis. Flowers 5-merous, heterostylous, the proximal flowers long-styled (co-sexual) and distal ones short-styled (functionally staminate), the plants andromonoecious. Calyx with the tube 4.3–7.5 mm long, 4–8 mm in diameter, shallowly cup-shaped to obconical, the lobes 4.5–11.3 mm long, triangular to somewhat lanceolate, sometimes varying in size in a single flower, densely bristly with trichomes like those of the stems, prickly or not. Corolla 3–3.6 cm in diameter, white, rotate, shallowly campanulate or tubular, lobed less than 1/8 of the way to the base, interpetalar tissue indistinguishable from the rest of the lobe or nearly so, copious and reaching nearly to the tips, the lobes 3.8–5 mm long, 16.5–25 mm wide, rounded to retuse, glabrous with pubescence restricted to the lobe apices on both surfaces, the trichomes minute, simple. Stamens equal; filament tube 1.5–1.7 mm long, glabrous; free portion of the filaments 1.4–1.8 mm long, glabrous; anthers 6.5–10 mm long, 2.2–3 mm wide, broadly lanceolate and tapering, connivent, glabrous, yellow, abaxially swollen in the lower half (gibbous) and somewhat papillate, poricidal at the tips, the pores directed distally, not elongating to slits with age. Ovary cup-shaped, moderately to densely pubescent, the trichomes simple, glandular, very tiny papillate and sessile to 0.6 mm long, 1–5-celled, thin-walled, the longer ones less abundant, pubescence sometimes restricted to the ovary apex; style 8.8–12 mm long in long-styled flowers, 1.2–3.6 mm long in short-styled flowers, straight, sparsely to moderately puberulent with very tiny papillate trichomes; stigma clavate, the surface minutely papillose. Fruit and seeds not known. Chromosome number not known.

##### Distribution

**Figure 10.**
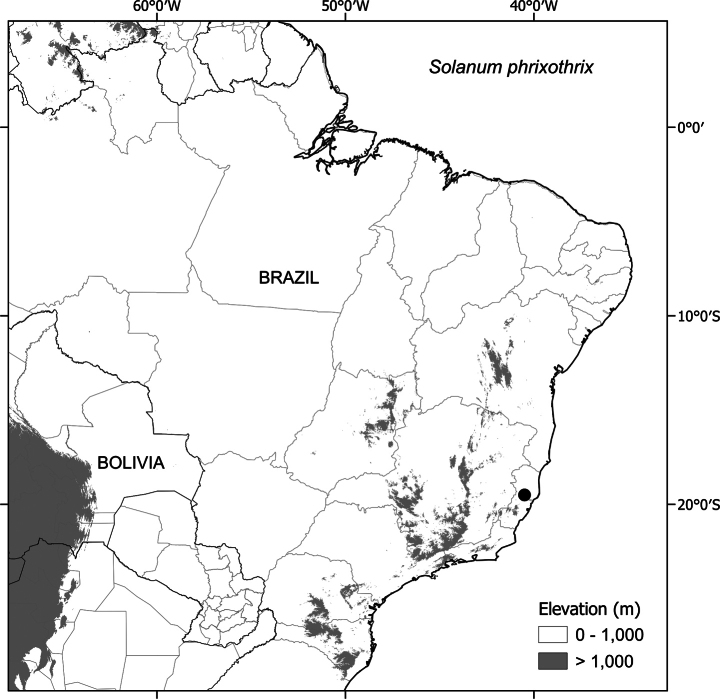
Distribution of *S.phrixothrix*.

(Fig. 10). *Solanumphrixothrix* is endemic to the south-eastern region of Brazil and known only from two collections; one with a specific locality from Espírito Santo State and another made by A. St.-Hilaire that is likely to be from Minas Gerais State (see Dwyer (1955)).

##### Ecology and habitat.

The only known collection with locality and vegetation type information of *S.phrixothrix* is from wet evergreen forests of the lower Rio Doce drainage, at approximately 13 m elevation. This collection is from a “cabruca” (cacao plantation). These cacao plantations retain the upper strata of the forest for shade, but the understorey is significantly damaged by shrub and herb removal, along with significant cacao leaf litter.

##### Common names and uses.

None recorded.

##### Etymology.

The species epithet is derived from the Greek, meaning with bristling (or horrid) hairs.

##### Preliminary conservation status

(IUCN 2020). *Solanumphrixothrix* is known from only two collections, gathered 200 years apart and so must have a preliminary assessment of Data Deficient. That said, however, it is imperative that more populations be sought to better assess its range and population density. It is likely to be of conservation concern, as the single collection with an accurate locality (*Folli 7506*) is from a highly disturbed anthropogenic site (cacao plantation), not within a protected area. It is near the Floresta Nacional de Goytacazes in Espiríto Santo State and should be sought there. Other protected areas close to this collection, such as Reserva Biológica de Sooretama and Reserva Natural Vale are well-inventoried and we have seen no specimens of *S.phrixothrix* from them in any of the many herbaria we consulted.

##### Discussion.

*Solanumphrixothrix* is a distinctive, densely bristly plant that has only been collected twice, once by Auguste St. Hilaire in the early 19^th^ century and more recently in 2017 (*Folli 7506*) along the Rio Doce in Espiríto Santo State. It differs from other taxa in the group in its rotate to rotate-pentagonal corollas (Fig. 9E, F) and densely bristly stems with no stellate trichomes present (Fig. 9B). The long inflorescences and somewhat delicate pedicels are similar to those of *S.aciculare*, but that species has copious glandular stellate to multangulate pubescence and stellate corollas (Fig. 2F, G, I). It is surprising that this species has escaped notice for so long, but members of the *S.hexandrum* group often occur in very small populations at the bases of rocky outcrops and may easily overlooked despite their large size and fearsome appearance.

*Solanumphrixothrix* shares dense bristly stems with *S.aciculare* but can be easily distinguished from that species by its lack of copiously glandular pubescence of long-stalked stellate trichomes. *Solanumphrixothrix* appears to lack long-stalked stellate trichomes on any part. White flowers are also found in *S.hydroides* (Fig. 7D) and *S.sublentum* (Fig. 13H), as well as occasionally in *S.aciculare* (Fig. 2F). Corollas in these three species are always stellate with variously deltate to triangular lobes, whereas those of *S.phrixothrix* are rotate and somewhat campanulate with the lobes reduced to tiny apiculae.

#### ﻿5. Solanum stagnale

Taxon classificationPlantaeSolanalesSolanaceae

Moric., Pl. Nouv. Amer. 34, tab. 23. 1837.

9297D1B3-4E22-5557-BFA5-600F9C216DD6

Fig. 11

Solanum moricandii Dunal, Prodr. [A. P. de Candolle] 13(1): 319. 1852, nom. illeg. superfl. Type: Based on Solanumstagnale Moric.
Solanum moricandii Dunal var. majus Dunal, Prodr. [A. P. de Candolle] 13(1): 319. 1852. Type: Based on Solanumstagnale Moric.
Solanum moricandii Dunal var. minus Dunal, Prodr. [A. P. de Candolle] 13(1): 320. 1852. Type; Brazil. Bahia: Ilheus, 1840, *J.S. Blanchet 3095A* (lectotype, designated by Whalen et al. (1981), pg. 69: G-DC [G00131269]; isolectotypes: G [G00343734], P [P00371688], W [acc. # 0004133]).
Solanum moricandii Dunal var. echinocalyx Dunal, Prodr. [A. P. de Candolle] 13(1): 320. 1852. Type: Brazil. Sin. loc., *J.J. Lalande s.n.* (holotype: P [P00371692]).
Solanum nolitangere Salzm. ex Dunal, Prodr. [A. P. de Candolle] 13(1): 320. 1852. Type: Brazil. Bahia: “in maritimis”, 1830, *P. Salzmann s.n.* (lectotype, designated by Whalen et al. (1981), pg. 69: G-DC [G00131246]; isolectotypes: C [C10019308], K [K000590187, K000590188], LE [LE00016989], P [P00366831, P00366832, P00366833, P00366834], W [acc. # 1889-0293774]).
Solanum nolitangere Dunal var. ochraceo-ferrugineum Dunal, Prodr. [A. P. de Candolle] 13(1): 320. 1852. Type: Brazil. Bahia: sin.loc., 1832, *J.S. Blanchet 710* (lectotype, designated by Whalen et al. (1981), pg. 69: G-DC [G00131245]).

##### Type.

Brazil. Bahia: sin. loc., *J.S. Blanchet 2085* (lectotype, designated by Whalen et al. (1981), pg. 69 [as holotype]: G [G00343733]; isolectotypes: BM [BM000617832], G-DC [G00131205], P [P00371689, P00578808, P00371690]).

##### Description.

Large, soft-wooded perennials 0.3 - several m tall. Stems terete, usually somewhat winged from the decurrent leaf bases, densely pubescent and sparsely prickly, the pubescence of whitish or reddish-cream porrect-stellate or occasionally multangulate (*Agra 617*) trichomes with multiseriate stalks 0.2–0.8 (-2.5) mm long, the lateral rays 4–9, 0.4–0.6 (-1) mm long, the mid-points much shorter than the rays, 0.05–0.2 mm long, the hairs densely interwoven and entirely concealing young stems, the prickles 1–2 cm long, 0.3–0.5 cm wide at the base, straight or recurved (e.g. *Blanchet 2085*) broad-based and strongly laterally compressed, often densely stellate-pubescent basally. Sympodial units difoliate, the leaves of a pair not geminate. Leaves shallowly lobed and coarsely repand; blades 9.5–30 cm long, 8–15 cm wide, ca. 1–1.5 times as long as wide, broadly elliptic to ovate, slightly discolorous, membranous or somewhat chartaceous, prickly on both surfaces along the veins with straight prickles to 15 mm long; adaxial surfaces densely pubescent with eglandular short- to long-stalked porrect stellate trichomes, the stalks 0.5–0.8 (-1) mm long, the rays 4–8, 0.5–0.7 mm long, the mid-points minute or equal in length to the rays, the lamina visible under the microscope; abaxial surfaces densely woolly-pubescent with stalked porrect stellate trichomes, the stalks 0.5–1 mm long, the rays 6–10, 0.5–1 mm long, often not in a single plane, the mid-points 0.2–0.4 mm long, much shorter than the rays; principal veins 4–5 pairs, with scattered straight prickles to 1.3 mm long, the prickles longer and larger on the mid-rib; base strongly decurrent along a winged petiole, the wing of laminar tissue to 0.5 cm wide on each side, often decurrent on to stem; margin lobed, the lobes 4–5, 1.5–3 cm long, 2–3 cm wide, deltate, acute- or round-tipped, often with irregular secondary lobing, the sinuses reaching less than halfway to the mid-rib ; apex acute or obtuse; petioles 0.8–5 cm, usually 1/4–1/3 the length of the blades and winged, stellate-pubescent like the stems, prickly. Inflorescences 2–7 cm long, extra-axillary or leaf-opposed, unbranched, with ca. 10 flowers, the axes densely stellate-pubescent, unarmed; peduncle 1–2 cm; pedicels 2–5 mm, 1–2.5 mm in diameter at the base, 2–2.6 mm diameter at the apex, articulated at the base; pedicel scars closely spaced 2–7 mm apart. Flowers 5-merous, heterostylous, with the lowermost long-styled (co-sexual) and the distalmost short-styled (functionally staminate), the plants probably andromonecious. Calyx with the tube 5–10 mm long, 8–10 mm in diameter, broadly obconical, the lobes 10–15 mm long, 5–8 mm wide, ovate-lanceolate or somewhat spathulate or tongue-shaped, obtuse or round apically, densely stellate-pubescent on both surfaces, often with scattered prickles on both surfaces near the mid-vein, often purple-tinged distally. Corolla 2.5–4.5 cm in diameter, white or lilac with a paler central star, stellate, lobed 2/3 to 3/4 of the way to the base, interpetalar tissue a thin edge on the lobes, the lobes 13- 20 mm long, 10–12 mm wide, ovate-lanceolate, densely stellate-pubescent abaxially, the trichomes with robust mid-points equal to or longer than the rays, glabrous adaxially, but the acute tips stellate-pubescent, the interpetalar tissue thin, glabrous. Stamens equal; filament tube minute; free portion of the filaments ca. 1 mm long, glabrous; anthers 7–10 mm long, ca. 3 mm wide, broadly lanceolate and tapering, connivent, glabrous, yellow, abaxially swollen in the lower half (gibbous) and somewhat papillate, poricidal at the tips, the pores directed distally, slightly extrorse, not elongating to slits with age. Ovary conical, densely stellate-pubescent, the trichomes with well-developed lateral rays; style 10–14 mm long, glabrous or sparsely stellate-pubescent in the lower half; stigma large and capitate. Fruit a globose to flattened-globose berry, 2–2.5 cm in diameter, whitish-green at maturity, sparsely stellate-pubescent, ultimately glabrous, the pericarp matte or slightly shiny; fruiting pedicels 0.5–1 cm long, usually less than 0.5 cm long, 3–5 mm in diameter at the base, ca. 6 mm in diameter at the apex; fruiting calyx only partially accrescent, tightly investing. but not completely covering fruit, the tube ca. 1.5 cm long, the lobes ca. 15–20 mm long, ca. 10 mm wide, not overlapping. Seeds ca. 100 per berry, ca. 2.5 mm long, ca. 1.5 mm wide, flattened reniform, dark brown, the surfaces minutely pitted, the testal cells pentagonal in outline. Chromosome number; 2n = 24 (Bernardello et al. 1994; voucher (grown in Indiana and, therefore, should be in IND, but not seen) *Carvahlo 3213*, possibly a misprint for *Carvalho 3219*).

**Figure 11.**
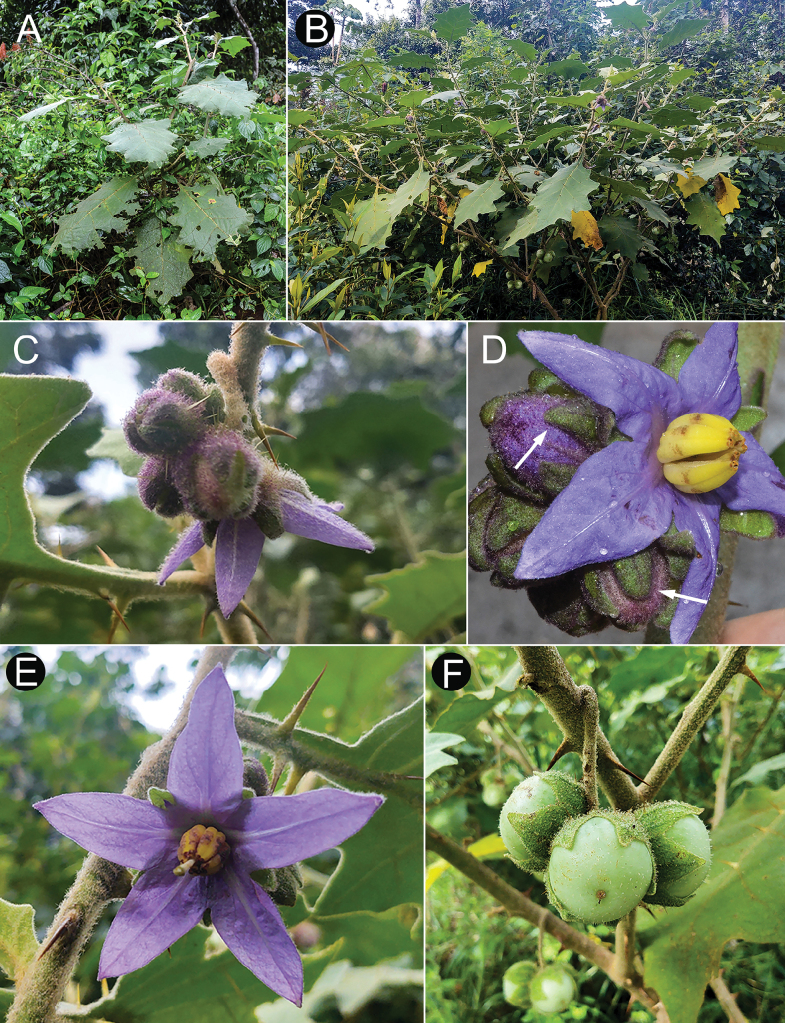
*Solanumstagnale***A** habit of a young plant **B** habit of an older reproductive plant **B, C** inflorescence congested with flowers clustered due to extremely short pedicels **D** flower buds and short-styled flower (arrows point to spathulate calyx lobes) **E** long-styled flower and leaf base markedly decurrent on to the petiole **F** berries (almost mature) with appressed, only partially accrescent fruiting calyces (**A**, **D***Giacomin & Stehmann 1930*, BHCB; **B**, **C**, **E**, **F** unvouchered field photograph, Bahia State [12°32'23"S, 38°03'08"W]). Photos: **A**, **D** Leandro L. Giacomin **B**, **C**, **E**, **F** Wagner Nogueira.

##### Distribution

**Figure 12.**
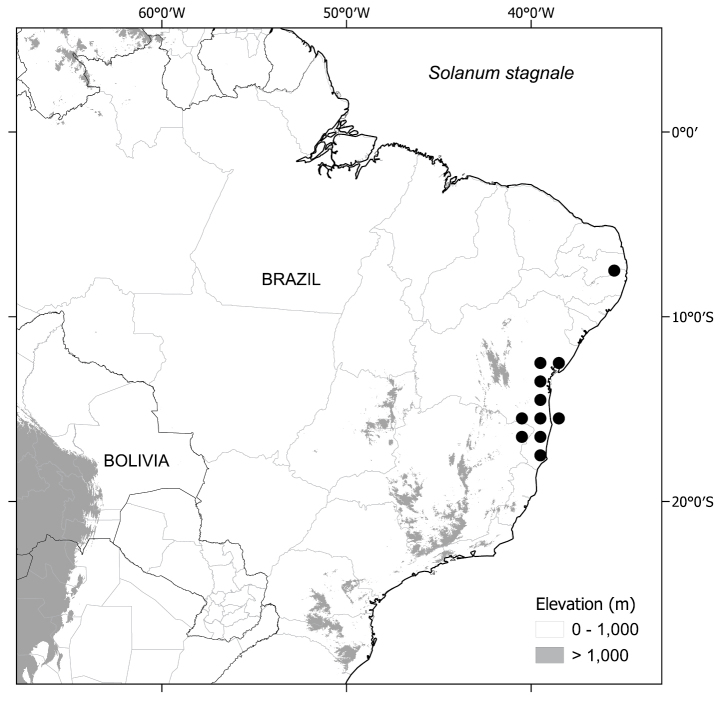
Distribution of *S.stagnale.*

(Fig. 12). *Solanumstagnale* is endemic to eastern Brazil; it has been recorded from the States of Bahia and adjacent northern Minas Gerais and disjunctly in Paraíba State. This disjunct distribution is unusual, the single collection from Paraíba is from the northern side of the São Franscisco River, the site of the Pernambuco area of endemism.

##### Ecology and habitat.

*Solanumstagnale* occurs in sandy coastal vegetation (restinga) habitat, in sand dunes, forests, forest edges and somewhat open habitats, from sea level to 300 m elevation.

##### Common names and uses.

None recorded.

##### Preliminary conservation status

(IUCN 2020). EOO (157,059 km^2^, LC); AOO (68 km^2^, EN). *Solanumstagnale* is known from more than five localities, even if the widely disjunct collection from Paraíba State is not included. Only one of these is within a protected area (Estação Ecológica de Cotegipe in Mun. Salvador, Bahia). The fragmented nature of the habitat and the absence of state or national level of protection for areas where it occurs suggests it should be assigned a preliminary conservation status of Vulnerable, based on criteria B 2 a,b i,ii,iii,iv.

##### Discussion.

Like all members of this group, *S.stagnale* has large, repand leaves. Most collections have strongly winged petioles with a wing extending fully to the base, but occasionally the wing becomes very narrow basally (*Rosas 1* from Salvador). The pedicels in both flower and fruit of *S.stagnale* are the shortest in the group, rarely reaching 5 mm long. *Solanumstagnale* is easily distinguished from *S.hexandrum*, with which it is most similar, by its pubescence of porrect-stellate trichome with usually more than 5 lateral rays, usually curved prickles, short, stubby pedicels usually less than 0.5 cm long, spathulate calyx lobes with rounded apices and berry that is not completely enclosed in an accrescent calyx. The trichomes of *S.stagnale* usually have mid-points that are shorter than or equal to the rays in length; in contrast, other species of the group have longer mid-points.

Whalen et al. (1981) treated *S.stagnale* as a member, albeit anomalous, of section Lasiocarpa (Dunal) D’Arcy and Whalen (1984) later included it in his equivalent *S.quitoense* Lam. species group. Both groups were composed of species, with the exception of *S.stagnale*, which are now recognised (Gagnon et al. 2022) as the Lasiocarpa clade; they share with members of the *S.hexandrum* group large repand leaves. Molecular data (Gouvêa 2020; Gagnon et al. 2022), however, clearly show that *S.stagnale* is related to *S.hexandrum* and other Brazilian endemic species of this group, not to the largely Andean members of the Lasiocarpa clade.

Dunal (1852) changed the name *S.stagnale* to *S.moricandii* because he felt it was inappropriate (“Blanchet in schedis non dicit hanc specimen crescere in stagnis, ut putat Moricand, et hâc ratione, nomen *stagnale* mutavi” – Blanchet does not say this specimen grows in ponds, as Moricand thinks, for this reason I have changed the name *stagnale*”: Dunal 1852: 319) rendering the name *S.moricandii* illegitimate and superfluous.

#### ﻿6. Solanum sublentum

Taxon classificationPlantaeSolanalesSolanaceae

Hiern., Kjoeb. Vidensk. Meddel. 1877–78: 53. 1878.

88E2C492-0A73-5F9C-AC6C-C74ADD79D5B1

Fig. 13

Solanum wettsteinianum Witasek, Denkschr. Kaiserl. Akad. Wiss. Wien, Math.-Naturwiss. Kl. 79 (advance separate): 50. 1910. Type. Brazil. São Paulo: prope “Fazenda Bella Vista” in districtu urbis S. Cruz ad flumen Rio Pardo, 500 m alt., Jul 1901, *R. von Wettstein s.n.* (lectotype, designated here: WU [acc. # 0038006]).

##### Type.

Brazil. Minas Gerais: Lagoa Santa, [24 Feb 1863], *E. Warming s.n.* (lectotype, designated here: C [C10019316]; possible isolectotype: [note date on sheet is 1866] S [acc. # S04-2985]).

##### Description.

Shrubs 1–3 m, erect or sometimes somewhat prostrate, armed. Stems terete, densely glandular-pubescent and sparsely prickly, the trichomes weak, simple uniseriate ca. 0.5 mm long with glandular tips, mixed with sparse short-stalked porrect stellate trichomes with 6–7 rays to 1 mm long, the mid-point to 1 mm long, gland-tipped or eglandular, the prickles 0.5–1.2 cm long, slightly to strongly curved and broad-based, ca. 0.5–1 cm in diameter at the base; new growth densely glandular pubescent with mixed simple and stellate trichomes like the stems; bark of older stems pale greyish-brown, somewhat glabrescent. Sympodial units difoliate, the leaves not geminate. Leaves shallowly lobed and repand, much smaller in younger branches; blades (5)8.5–17 cm long, (3)6–13 cm wide, ca. 1.3–1.6 times as long as wide, broadly elliptic or ovate, widest at or just below the middle, membranous, concolorous, sparsely prickly on both surfaces along the veins with straight prickles 0.3–1 cm long; adaxial surface densely pubescent with a mix of short-stalked porrect stellate trichomes with 5–7 rays ca. 1 mm long, trichomes consisting of solely unicellular or multicellular gland-tipped mid-points to 1.2 mm long (probably derived from stellate trichomes) and sessile, papillate glands composed of 4 cells; abaxial surface pubescent like the adaxial surface, but lacking the sessile papillate glands, also with delicate sessile porrect stellate trichomes with 4–5 rays ca. 0.3 mm long and mid-points shorter than the rays, these underneath the dense layer of larger short-stalked trichomes; principal veins 4–5 pairs, usually sparsely prickly on both surfaces with straight prickles to 0.9 cm long; base somewhat cordate-angular to hastate or sagittate-hastate from the basiscopically directed lowest leaf lobes, occasionally acute to abruptly attenuate, usually not decurrent on to the petiole; margins shallowly and broadly lobed, the lobes 4–5, 1–2.5 long, 2–4 cm wide, apically acute to acuminate, sometimes minutely secondarily lobed, the sinuses less than 1/4 of the way to the mid-rib; apex acute to acuminate; petiole (1-) 1.5–7 cm long, prickly with straight prickles to 1 cm long, densely glandular pubescent like the stems with a mix of simple trichomes apparently consisting of unicellular or multicellular mid-points with glandular tips and sparse porrect stellate trichomes. Inflorescences internodal, 2–9 cm long, unbranched, with 3–6 flowers. but only one open at a time; axes densely glandular pubescent like the stems with a mix of unicellular and multicellular gland-tipped simple uniseriate trichomes (derived from mid-points of stellate trichomes) and sparse porrect stellate trichomes with glandular mid-points; peduncle 1.5–5 cm long; pedicels 1.2–1.5 cm long, ca. 1 mm in diameter at the base, ca. 2.5 mm in diameter at the apex (excluding trichomes), erect to spreading, densely glandular pubescent like the inflorescence axes and stems and occasionally with a few straight prickles, articulated at the base; pedicel scars more or less evenly spaced 4–5 mm apart, further apart in fruit, distally and, in young inflorescences, more tightly packed. Buds globose to ovoid, the corolla ca. halfway exerted from the calyx tube just before anthesis. Flowers 5-merous, co-sexual or perhaps a few distal flowers short-styled and functionally staminate, the plants only weakly andromonoecious. Calyx with the tube 3.5–4 mm long, 5–6 mm in diameter, deeply to shallowly broadly cup-shaped, plicate from the fused bases of adjacent lobes, usually invaginate at the base, the lobes 7–10 mm long, 2.3–5 mm wide, long-triangular, apically acuminate, densely glandular pubescent with a mix of simple uniseriate (mid-points?) and sparse short-stalked or sessile stellate trichomes, often with a few straight prickles 0.2–1.5 mm long on the main veins abaxially. Corolla 3.5–5 cm in diameter, purple to pale violet or white, shallowly stellate, lobed ca. 1/4 of the way to the base, interpetalar tissue thin, glabrous, the lobes 9–12 mm long, 11–19 mm wide, spreading to slightly cupped, densely pubescent abaxially where exposed in bud with short-stalked and sessile stellate trichomes, these occasionally glandular, glabrous adaxially, but occasionally with a few minute prickles along the veins. Stamens equal; filament tube minute; free portion of the filaments 0.5–1 mm long, glabrous; anthers 8.5–9 mm long, 2.6–3 mm wide, broadly lanceolate and tapering, connivent, glabrous, yellow, abaxially swollen in the lower half (gibbous) and somewhat papillate, poricidal at the tips, the pores directed distally, not elongating to slits with age. Ovary conical, glabrous; style 14–16 mm long, glabrous, widening markedly distally; stigma clavate or broadly capitate, the surface minutely papillate. Fruit a globose berry, 1.4–2 cm in diameter, green or pale whitish-green, glabrous, the pericarp matte when dry, opaque, the berry completely enclosed in the accrescent saccate calyx; fruiting pedicel 1.6–2.5 cm long, 1–1.5 mm in diameter at the base, 3–5 mm in diameter at the apex, spreading or pendent from the weight of the fruit; fruiting calyx strongly accrescent, inflated and invaginate, exceeding the length of the berry, but not completely enclosing it, the tube 1.5–2 cm long, saccate (invaginate) at the base, the lobes 0.8–0.9 cm long, often broken in dried specimens, not overlapping, densely glandular pubescent and occasionally prickly like the calyx in flower. Seeds 80–100 per berry, ca. 2.5 mm long, ca. 2 mm wide, flattened reniform, reddish-brown when dry, the surface minutely pitted, the testal cells thick-walled and sinuate in outline. Chromosome number not known.

**Figure 13.**
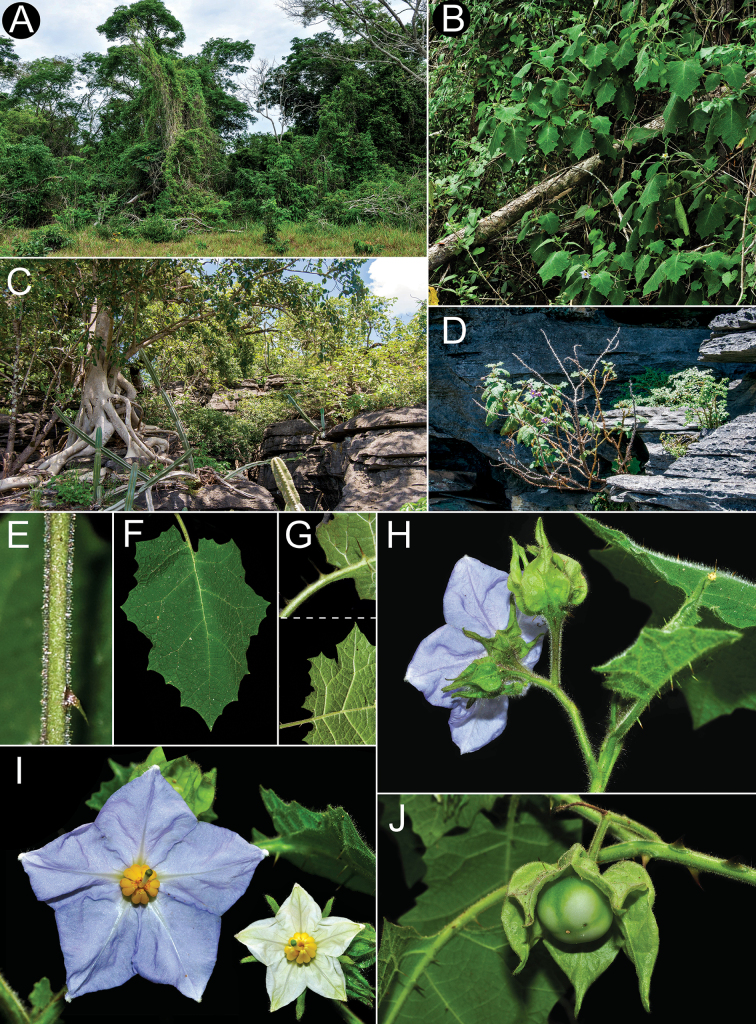
*Solanumsublentum***A** habitat of arboreal restinga (coastal scrub-forest transition) forest edges **B** habit **C** habitat in seasonally dry deciduous forests **D** habit in rocky inselberg **E** stem with recurved prickles and unbranched glandular pubescence **F** leaf with cordate base and secondary lobing **G** variation in leaf bases and lobing **H** inflorescence **I** long-styled flowers showing colour polymorphism **J** mature berry with accrescent invaginate calyx (**B**, **F, H–J***Gouvêa & Guerrero* 452; **D***Stehmann et al. 6370*; **E, G***Stehmann et al. 6372*). Photos: **A**, **B**, **H–J** Yuri F. Gouvêa **C**, **D, E, G** João R. Stehmann.

##### Distribution

**Figure 14.**
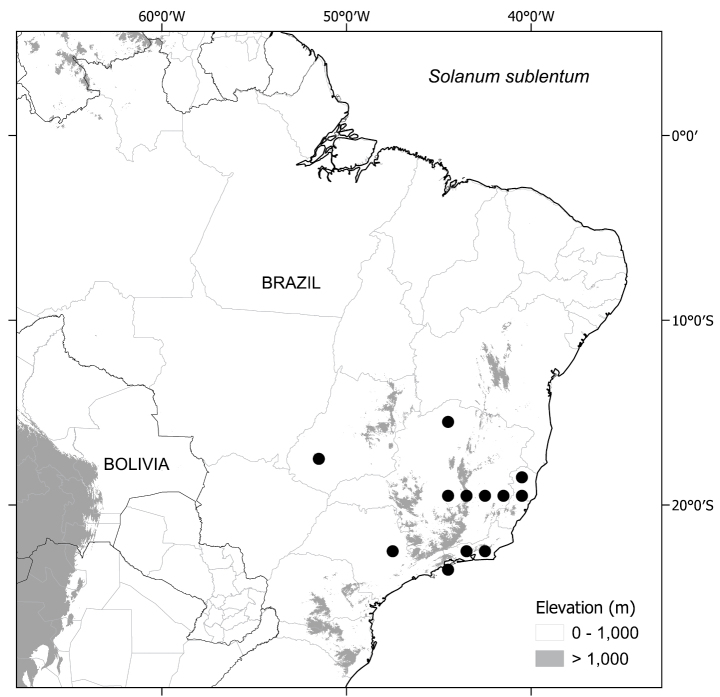
Distribution of *S.sublentum.*

(Fig. 14). *Solanumsublentum* is recorded to south-eastern and central Brazil, in the States of Espírito Santo, Goiás, Minas Gerais, Rio de Janeiro and São Paulo. The collection from Goiás is discontinuous from the rest of the species range (see discussion).

##### Ecology and habitat.

*Solanumsublentum* occupies primarily forest understory, edges and clearings of wet coastal and semi-deciduous forests in the Atlantic Forest domain (Mata Atlântica; Fig. 13A, B), as well as semi-deciduous and deciduous seasonally dry tropical forests (Fig. 13C, D) in ecotonal zones or within savannah matrices in the Cerrado domain. The forests where S.sublentum occurs can be associated with granite/gneiss (wet and semi-deciduous forests in coastal and sub-coastal regions) and limestone or basaltic (inland seasonally dry tropical forests in savannah matrices) outcrops, it is the only species in the group and one of the few American species of *Solanum* found in STDFs associated with limestone outcrops. *Solanumsublentum* grows both in deep soils and in rock cavities, in fissures and in small, shallow soil patches that accumulate on bare rocks (Fig. 13D); from sea level to 800 m elevation.

##### Common names and uses.

None recorded.

##### Preliminary conservation status

(IUCN 2020). EOO (642,872 km^2^, LC); AOO (72 km^2^, EN). *Solanumsublentum* is known from eight localities (including the extremely disjunct occurrence in the State of Goiás), at least three of which are in protected areas (Parque Nacional Cavernas do Peruaçu, Estação Biológica de Caratinga in Minas Gerais State; Parque Municipal Sombra da Tarde in Espirito Santo State) with other collections from the mountains near Rio de Janeiro in what is now Parque Nacional da Serra dos Orgãos (e.g. “Organ Mount”). Even with the disjunct occurrence in Goiás excluded, *S.sublentum* has the largest extent of occurrence of any of the species of the *S.hexandrum* group. Nevertheless, due to the extreme level of habitat alteration in the region where it occurs and the paucity of recent collections (most of those used in calculating the EOO are more than a century old), we consider it of some conservation concern and suggest a preliminary status of Near Threatened, based on criteria B 2 a,b ii,iii, iv.

##### Discussion.

*Solanumsublentum* is easily distinguishable from other members of this group in its markedly plicate accrescent calyces that exceed, but do not completely enclose the berries and simple, glandular pubescence with a mix of much less abundant stellate trichomes.

The majority of trichomes of *S.sublentum* are simple, uniseriate and unicellular or multicellular. Their shape and overall morphology suggest they are structurally analogous to mid-points of stellate trichomes without rays, like those that occur in the Acanthophora clade (Nee 1979; Hilgenhof et al. 2023) and most members of the Gonatotrichum group of the Brevantherum clade (Stern et al. 2013).

The single collection of *S.sublentum* from Goiás (*Hatschbach 34747*) is from Mun. Jataí, in the extreme southwest of the State. The habitat is stated as “clareiras da mata” (forest clearings) and the area in which it was collected was a remnant of the now very restricted Atlantic Forest. The southernmost portion of Goiás State is the original limit of the Atlantic Rainforest domain in central Brazil and an ecotonal zone with the Cerrado domain, where contiguous forested formations were found in the past (IBGE 2012). These mostly semi-deciduous forests were either considered part of the Cerrado (“Cerradão”) or the Atlantic Rainforest itself. They have been largely converted first to cattle farming and later to soy and corn plantations (Oliveira 2007). No recent collections from this locality have been seen, so recollection there is a priority.

*Solanumsublentum* is similar to *S.aciculare* in possessing glandular indument and strongly accrescent, inflated and invaginate fruiting calyx lobes. The pubescence of *S.sublentum* is of unbranched trichomes (the bases and mid-points of modified stellate trichomes), while that of *S.aciculare* is of long-stalked stellate trichomes with glands on each ray tip. Prickles in *S.sublentum* are sparse and recurved, whereas those of *S.aciculare* are straight and denser especially on stems. The calyx completely covers the berry in *S.aciculare* (Fig. 2H) while, in *S.sublentum*, the berry is clearly visible (Fig. 13J).

Many different collecting dates are written on the Warming collections at C used by Hiern (1878) to describe *S.sublentum.* We have chosen the only sheet with both flowers and fruits (C10010316), showing the characteristic saccate calyx in fruit of *S.sublentum.* The many syntype collections in Copenhagen and sent elsewhere by Hiern have a confusing panoply of dates and some may be isolectotypes, but, in the absence of unambiguous dates on these sheets, we do not recognise them as such. The sheet we here cite as a possible isolectotype at S (acc. # S04-2985) has a label stating 1866, so it too is likely not a duplicate, but it is the only original material we have seen outside of Copenhagen.

### ﻿“Names” (designations) not validly published

*Solanumminax* Mart. ex Sendtn., Fl. Bras. (Martius) 10: 71. 1846. Not intended as a new name, cited as a manuscript name under *Solanumhexandrum* Vell. “β minax” = *S.hexandrum*

*Solanummultiangulatum* Vell., Fl. Flumin. 91. 1829 [1825], nom. utique rej. = *S.hexandrum* (as = *S.echidnaeforme* Dunal in Knapp et al. 2015).

*Solanumtubiflorum* Dunal, Prodr. [A. P. de Candolle] 13(1): 318. 1852, pro syn. *Solanummaroniense* Poit. = *S.hexandrum* (herbarium name on *Sellow s.n.* in BM [BM000935474]).

## Supplementary Material

XML Treatment for Solanum aciculare

XML Treatment for Solanum hexandrum

XML Treatment for Solanum hydroides

XML Treatment for Solanum phrixothrix

XML Treatment for Solanum stagnale

XML Treatment for Solanum sublentum

## ﻿Additional information

### Author ORCIDs

Sandra Knapp https://orcid.org/0000-0001-7698-3945

Yuri F. Gouvêa https://orcid.org/0000-0002-7162-8458

Leandro L. Giacomin https://orcid.org/0000-0001-8862-4042

### Data availability

All of the data that support the findings of this study are available in the main text or Supplementary Information. Supplementary files can also be found on the Natural History Museum’s Data Portal (https://doi.org/10.5519/vv8f8pkx).

## ﻿Appendix 1

**Index to numbered collections**

For collections made by two or more collectors, only primary (first listed) collector is presented here. Collections by anonymous collectors without date or other identifying features are not listed. Full collector strings can be found in the Suppl. materials 1, 2. This index and the searchable files are also available on the NHM Data Portal (https://doi.org/10.5519/xuvrw79j).

Agra, MF 617 (*stagnale*); 7216, 7234, 7254, 7359 (*hexandrum*).

Aguiar, MIH 446 (*hexandrum*).

Aleixo, S sp28-1 (*hexandrum*).

Almeida-Lafetá, RC 78 (*hexandrum*).

Amorim, AM 2706 (*stagnale*); 7124 (*hexandrum*).

Andrade, IR 147 (*hexandrum*).

Araujo, D 801, 1399 (*hexandrum*).

Arbo, MM 7792 (*sublentum*).

Arbocz, GF 160, 1446 (*hexandrum*).

Azevedo, IFP 28 (*hexandrum*).

Bandeira, BC 147 (*hexandrum*).

Barbosa, MR 1508 (*hexandrum*).

Barth, OM P-11 (*hexandrum*).

Belém, RP 1078 (*stagnale*).

Bernardi, L. 32 (*hexandrum*).

Blanchet, JS 32, 179, 370, 710, 1843, 2085, 3095A, 3095 (*stagnale*).

Boechat, SL 17 (*hexandrum*).

Bovini, MG 658, 1072, 2128 (*hexandrum*).

Brade, AC 11018 (*sublentum*) 18190, 18312 (*hexandrum*).

Braga, JMA 677, 874, 1451, 6244 (*hexandrum*).

Brotto, ML 3269 (*hexandrum*).

Bünger, MO 531 (*hexandrum*).

Burchell, WJ 1157 (*sublentum*).

Callejas, R 1582 (*stagnale*).

Campos Porto, P 776 (*hexandrum*).

Campos, WG 55 (*hexandrum*).

Campos, MTVA 150 (*hexandrum*).

Carauta, JPP 2348, 2780, 2803, 3282, 4592, 5330 (*hexandrum*).

Cardoso, LJT 694, 1382 (*hexandrum*).

Carrijo, TT 1490, 1827, 2015 (*hexandrum*).

Carvalho, AM de 452, 1490, 3219 (*stagnale*); 6866 (*hexandrum*).

Castellar, A 6, 14 (*hexandrum*).

Ceccantini, GCT 2812 (*sublentum*).

Clarissa, C 3 (*hexandrum*).

Colletta, GD 166 (*hexandrum*).

Cordeiro, J 6030 (*hexandrum*).

Costa, APL 16985 (*hexandrum*).

Costa, IG 543 (*hexandrum*).

Costa, LV 89 (*sublentum*); 199 (*hexandrum*); 200 (*sublentum*); 865 (*hexandrum*).

Couto, DR 690, 1661 (*hexandrum*).

Davis, PH D. 59826 (*hexandrum*).

Demuner, V 2922, 4131, 4944 (*hexandrum*).

Duarte de Barros, W 1121 (*hexandrum*).

Duarte, AP 1573 (*hexandrum*); 5002 (*sublentum*).

Duarte, C 8 (*hexandrum*).

Dusén, P 5135 (*hexandrum*).

Egler, W 105 (*hexandrum*).

Eiten, G 7914 (*hexandrum*).

Emygdio, L RB- 38680 (*hexandrum*).

Erickson, HT 1 (*hexandrum*).

Esteves, GL 2634 (*hexandrum*).

Faria, ALA 120 (*hexandrum*).

Farney, C 161 (*hexandrum*).

Ferreira, LA 69108 (*hexandrum*).

Fiaschi, P 1987 (*hexandrum*).

Flores, TB 1465, 1705, 1717 (*hexandrum*).

Folli, DA 1943 (*stagnale*); 7560 (*phrixothrix*).

Fontana, AP 3051, 5440 (*hexandrum*).

Forzza, RC 5029, 5125, 8803 (*hexandrum*).

Fraga, CN 1899 (*hydroides*); 2012 (*hexandrum*).

França, GS 348, 548 (*hexandrum*).

Freire de Carvalho, L d’A 559, 662 (*hexandrum*).

Freitas, L 587 (*hexandrum*).

Frutuoso, LCF 108 (*hexandrum*).

Furlan, A 1445 (*hexandrum*).

Galland, Y 13 (*hexandrum*).

Gardner, G 533 (*hexandrum*); 799 (*sublentum*); 800 (*hexandrum*).

Gaudichaud, C 500, 501 (*hexandrum*).

Gemtchújnicov, ID de 250 (*hexandrum*).

Gentry, AH 49340 (*hexandrum*).

Giacomin, LL 499 (*sublentum*); 875, 1689, 1827, 1833, 1844 (*hexandrum*); 1930 (*stagnale*).

Giordano, LC 392, 517 (*hexandrum*).

Glaziou, AFM 338 (*sublentum*); 3777, 5960, 8848 (*hexandrum*); 8879 (*sublentum*); 13082 (*hexandrum*).

Glocker, EF von 42, 102 (*stagnale*).

Góes, OC 228, 976, 1019, 1181 (*hexandrum*).

Goldenberg, E 32388 (*hexandrum*).

Gouvêa, YF 102 (*aciculare*); 135, 137, 158, 159 (*hexandrum*); 280, 281, 282, 283, 284 (*aciculare*); 492 (*hydroides*).

Guedes, ML 3649, 6553 (*stagnale*); 19611 (*sublentum*).

Harley, RM 17932 (*stagnale*).

Hatschbach, GG 34747 (*sublentum*); 46667 (*hexandrum*); 47806 (*aciculare*); 48677, 57934 (*hexandrum*); 62938 (*hydroides*); 63105 (*stagnale*).

Herb. Richard 533 (*hexandrum*).

Heringer, EP 879 (*hexandrum*); 6455 (*sublentum*).

Hoehne, FC SP-42651, SP-42653 (*hexandrum*).

Horst, MIA 16, 150 (*hexandrum*).

Hottz, D 290 (*hexandrum*).

Ichaso, CLF 154 (*hexandrum*).

Irwin, HS 2076 (*hexandrum*).

Isern, J 6467 (*hexandrum*).

Jardim, JG 1743 (*hexandrum*); 3151 (*aciculare*).

Jascone, CES 1111 (*hexandrum*).

Jouvin, PP 468 (*hexandrum*).

Kirizawa, M 1889 (*hexandrum*).

Kollmann, L 3459 (*sublentum*); 10314 (*hexandrum*); 11385 (*hydroides*).

Kreiger, L (*hexandrum*).

Krieger, L (Padre) 1073, 7453, 7515, 8845, 11769, 13395 (*hexandrum*).

Kuhlmann, M 2678 (*hexandrum*).

Kuntz, J 645 (*hexandrum*).

Leitão Filho, HF 1373, 1379 (*hexandrum*).

Leoni, LS 524, 3116, 7341, 7345, 7346 (*hexandrum*).

Liene, D 3912 (*hexandrum*).

Lima, JR 35, 39, 42, 83 (*hexandrum*).

Lira Neto, JA 164, 648 (*hexandrum*).

Lobão, A 1676 (*hexandrum*).

Loefgren, A CGG-1868, 3129 (*hexandrum*).

Lombardi, JA 1280 (*hexandrum*); 1814, 2355 (*sublentum*); 2388, 3120 (*hexandrum*); 5076, 5326 (*stagnale*); 8215, 8951 (*hexandrum*).

Lopes, MA 417 (*sublentum*).

Luber, J 101 (*hydroides*).

Lucas, EJ 625 (*hexandrum*).

Lund, PW 621 (*sublentum*).

Machado, TM 298 (*hexandrum*); 673 (*hydroides*).

Magalhães, MG 17651 (*aciculare*).

Magnago, LFS 467 (*hexandrum*); 579 (*sublentum*).

Manhães, VC 172 (*hexandrum*).

Mantovani, W 138 (*hexandrum*).

Marcolino, F 152 (*hexandrum*).

Marquete Ferreira da Silva, N 93, 242 (*hexandrum*).

Marquete, R 802 (*hexandrum*); 1092 (*sublentum*).

Martinelli, G 13, 3558, 8860 (*hexandrum*).

Martius, CFP 253 (*sublentum*).

Mattos Silva, LA 833 (*stagnale*); 4152 (*aciculare*).

Mattos, J 15755 (*hexandrum*).

Mauad, L.P. 6 (*hexandrum*).

Mautone, L 274, 422, 1356 (*hexandrum*).

Mello-Silva, R 1727 (*hexandrum*).

Mexia, Y 4118, 4731, 5033 (*hexandrum*).

Miers, J 2731 (*hexandrum*); 3601, 3656 (*sublentum*).

Mori, SA 10459 (*aciculare*); 14066 (*stagnale*).

Mosén, CWH 2540 (*hexandrum*).

Moura, R 1198 (*hexandrum*).

Nadruz, M 2795 (*hexandrum*).

Nardin, CF 40 (*hexandrum*).

Nee, M 3373 (*sublentum*).

Neves, PT 45 (*hexandrum*).

Oliveira, A 1107 (*hexandrum*).

Oliveira, CAL de 261 (*hexandrum*).

Oliveira, SA 1 (*hexandrum*).

Ostenfeld, CH 5560 (*hexandrum*).

Pabst, G 5216 (*hexandrum*).

Paula, LFA de 148, 247, 388, 581, 669 (*hydroides*); 972, 1180 (*aciculare*).

Pedroni, F 2454 (*hexandrum*).

Pereira, E 1258, 2271, 3912 (*hexandrum*).

Pereira, FB 45/ 35 (*hexandrum*).

Pereira, LA 1557 (*hexandrum*).

Pereira, OJ 829 (*hexandrum*); 6545 (*hydroides*).

Pinheiro, RS 2115 (*aciculare*).

Pinto, HV 496 (*hydroides*).

Pinto, LJS 246 (*hexandrum*).

Pirani, JR 803, 1032 (*hexandrum*).

Pizziolo, W 119 (*sublentum*).

Plowman, TC 2753 (*sublentum*).

Pohl, JBE 108 (*hexandrum*); 5488 (*sublentum*).

Ponte, ACE 29801 (*hexandrum*).

Queiroz, LP de 15302 (*aciculare*).

Queiroz de Melo 63 (*hexandrum*).

Quinet, A 23/55, 97, 32/140 (*hexandrum*).

Raben, FC 19 (*sublentum*); 308 (*sublentum*); 310 (*hexandrum*).

Ramalho, RS 1190, 1255, 1309 (*hexandrum*).

Regnell, AF Rio-164, Rio-342 (*sublentum*).

Reidel, L 32 (*sublentum*); 197 (*stagnale*).

Rosa, M 213 (*hexandrum*).

Rosa, P 1017 (*hexandrum*).

Saint-Hilaire, A de A1-393 (*sublentum*); A1-379, B-13 (*hexandrum*); B1-1046 (*phrixothrix*); B2-33, C-48 (*hexandrum*).

Salgado, CS 79 (*hexandrum*).

Salino, A 3259 (*sublentum*); 3513, 4112, 5885, 14662 (*hexandrum*).

Sampiao, AJ 3340 A (*hexandrum*).

Santos, HGP dos 352 (*hexandrum*).

Santos, MCF dos 1966 (*hexandrum*).

Schott, HW 5439 (*sublentum*); 5444 (*hexandrum*).

Schüch, G 5438 (*hexandrum*).

Sellow, F 70, 120 (*hexandrum*); 120[a] (*sublentum*).

Sellow, F (*hexandrum*).

Sellow, F Silva, JM 58677 (*hexandrum*).

Sobral, M 6769 (*stagnale*); 8250 (*hexandrum*).

Souza, JP 641 (*hexandrum*).

Souza, TP 16 (*hexandrum*).

Stehmann, JR 3839 (*stagnale*); 4513 (*hexandrum*); 6370, 6372 (*sublentum*); 6387 (*aciculare*).

Sucre, D 6245, 7487, 8923 (*hexandrum*); 9668 (*sublentum*).

Tsuji, R 1119 (*hexandrum*).

Vasconcellos Neto, J 6625 (*sublentum*; 9265 (*hexandrum*).

Verdi, M 3136 (*hexandrum*).

Vermelho 18 (*hexandrum*).

Vervloet, RR 3372 (*hexandrum*).

Vidal, MRR 259 (*hexandrum*).

Vieira, MF 406 (*hexandrum*).

Vimercat, JM 239 (*hexandrum*).

Vinha, PC 1380 (*hexandrum*).

Völtz, RR 2289, 2549a (*hexandrum*).

Webster, GL 25426 (*sublentum*).

Weddell, HA 435, 678 (*hexandrum*).

Weyland, MC 333 (*hexandrum*).

Widgren, JF 141 (*hexandrum*).

Wied-Neuwied, M 1[25], 4 (*hexandrum*).

Without collector 156, 12010 (*hexandrum*).

## Supplementary materials

Supplementary material 1 All specimens of members of the *Solanumhexandrum* group examined Data type xlsx https://binary.pensoft.net/file/1278595This dataset is made available under the Open Database License (http://opendatacommons.org/licenses/odbl/1.0/). The Open Database License (ODbL) is a license agreement intended to allow users to freely share, modify, and use this Dataset while maintaining this same freedom for others, provided that the original source and author(s) are credited.Sandra Knapp, Yuri F. Gouvêa, Leandro L. Giacomin

Supplementary material 2 All collection events of members of the *Solanumhexandrum* group seen Data type xlsx https://binary.pensoft.net/file/1278596This dataset is made available under the Open Database License (http://opendatacommons.org/licenses/odbl/1.0/). The Open Database License (ODbL) is a license agreement intended to allow users to freely share, modify, and use this Dataset while maintaining this same freedom for others, provided that the original source and author(s) are credited.Sandra Knapp, Yuri F. Gouvêa, Leandro L. Giacomin
